# Shape Reconstruction Processes for Interventional Application Devices: State of the Art, Progress, and Future Directions

**DOI:** 10.3389/frobt.2021.758411

**Published:** 2021-11-19

**Authors:** Sujit Kumar Sahu, Canberk Sozer, Benoit Rosa, Izadyar Tamadon, Pierre Renaud, Arianna Menciassi

**Affiliations:** ^1^ The BioRobotics Institute, Scuola Superiore Sant’Anna, Pisa, Italy; ^2^ Department of Excellence in Robotics & AI, Scuola Superiore Sant’Anna, Pisa, Italy; ^3^ ICube, CNRS, INSA Strasbourg, University of Strasbourg, Strasbourg, France

**Keywords:** shape reconstruction, electromagnetic sensor, optical sensors, medical image based techniques, passive stretchable sensors, sensing in minimally invasive surgery

## Abstract

Soft and continuum robots are transforming medical interventions thanks to their flexibility, miniaturization, and multidirectional movement abilities. Although flexibility enables reaching targets in unstructured and dynamic environments, it also creates challenges for control, especially due to interactions with the anatomy. Thus, in recent years lots of efforts have been devoted for the development of shape reconstruction methods, with the advancement of different kinematic models, sensors, and imaging techniques. These methods can increase the performance of the control action as well as provide the tip position of robotic manipulators relative to the anatomy. Each method, however, has its advantages and disadvantages and can be worthwhile in different situations. For example, electromagnetic (EM) and Fiber Bragg Grating (FBG) sensor-based shape reconstruction methods can be used in small-scale robots due to their advantages thanks to miniaturization, fast response, and high sensitivity. Yet, the problem of electromagnetic interference in the case of EM sensors, and poor response to high strains in the case of FBG sensors need to be considered. To help the reader make a suitable choice, this paper presents a review of recent progress on shape reconstruction methods, based on a systematic literature search, excluding pure kinematic models. Methods are classified into two categories. First, sensor-based techniques are presented that discuss the use of various sensors such as FBG, EM, and passive stretchable sensors for reconstructing the shape of the robots. Second, imaging-based methods are discussed that utilize images from different imaging systems such as fluoroscopy, endoscopy cameras, and ultrasound for the shape reconstruction process. The applicability, benefits, and limitations of each method are discussed. Finally, the paper draws some future promising directions for the enhancement of the shape reconstruction methods by discussing open questions and alternative methods.

## Introduction

Over the past decade, robot-assisted minimally invasive procedures (MIP) have gained much momentum to improve the traditional surgical approaches. They have revolutionized the way clinician performs complex surgeries and diagnoses. These procedures involve different complex techniques, such as colonoscopy, ureteroscopy, laparoscopy, thoracoscopy, etc., in which specific tasks are performed to get access to body anatomy. In order to achieve such complicated tasks, various continuum flexible robotic systems such as concentric tubular robots (Gafford et al., 2020; Dupont et al., 2009; Girerd et al., 2017), cable-driven robots (Tamadon et al., 2018; Abdelaziz et al., 2012; Kutzer et al., 2011), soft robots (Cianchetti et al., 2014; De Falco et al., 2017; Ranzani et al., 2015), catheters (Murai et al., 2018; Fu et al., 2011; Iacovacci et al., 2018), flexible needles (Culmone et al., 2017; Okazawa et al., 2005; Engh et al., 2006), fluid-operated robots (Shiva et al., 2016; Sozer et al., 2020; Ranzani et al., 2016), and shape memory alloy manipulators (Shi et al., 2014a; Tung et al., 2007; Abdul Kadir et al., 2019) are continuously developed. Many research groups also discussed different robotic systems according to their actuation strategy (Zhong et al., 2020), control (Chikhaoui and Burgner-Kahrs, 2018), and autonomous capabilities (Haidegger, 2019). These manipulators can take any shape in space by performing bending, extension, contraction, and torsion of their structural components. These features help them to reach the target in a dexterous way when in confined environments, by following a complicated path through the body lumen. Their flexibility and adaptability enhance the capabilities of surgeons to carry out different surgical or diagnostic procedures. Due to the capabilities of these manipulators in terms of locomotion, manipulation, and compliance, they produce significant benefits such as less post-operative complication morbidity, reduced intraoperative blood loss, and shorter hospital stay.

While continuum manipulators furnish substantial advantages, they have certain limitations too. Though they can adapt to numerous shapes with various curvatures, difficulties are encountered during their active control. The numerous shape changes may damage surrounding healthy tissues due to unexpected interactions. Thus, they need to be guided carefully to avoid anatomical obstacles and sensitive tissues (Wei et al., 2017). Sometimes the backbone information is also difficult to control in presence of external forces acting on the manipulators. Therefore, the pose and shape information of the robot is needed in real-time to reduce injury during surgery. It will also help to perform accurate maneuvering by providing feedback to the controller.

In the framework of the shape reconstruction process, simple shape modeling approaches for continuum robots based on their kinematics have been evolved tremendously for determining the shape of the flexible robots (Bailly and Amirat, 2005; Rucker et al., 2010; Webster and Jones, 2010; Li et al., 2014). These methods of calculating the shape and backbone deformation of robots rely on the accuracy of models and consider piecewise constant curvature assumptions. Therefore, they may become invalid when the robots are affected by external loads. More comprehensive approaches are proposed based on Cosserat rod theory combined with static model (Rucker and Webster, 2011), (Jones et al., 2009), (Trivedi et al., 2008) and elliptical integral considering a known payload (Xu and Simaan, 2009) to achieve more accurate shape estimation. Another approach, such as model-based Rayleigh-Ritz formulation (Roesthuis et al., 2012) was also developed to accurately estimate the shape of the medical robots. Regardless of the improvement of model-based shape estimation to increase their robustness, it is however very challenging to implement in real-time applications under unknown payloads.

On the other hand, sensor-based shape reconstruction methods are more practical and show acceptable accuracy and consistency in measuring the real-time shape in free space as well as the unknown environments. For example, Fiber Bragg Grating (FBG) sensors are studied and developed to reconstruct the shape of manipulators. They show many benefits, such as short response time, miniature size, biocompatibility, non-toxicity, and high sensitivity (Roesthuis et al., 2013), (Ryu and Dupont, 2014). In these methods, several FBG sensors which can measure axial strain are arranged along the length of a robot to calculate the curvature. Afterward, the shape of the manipulator is reconstructed using the curvature information. Recently, electromagnetic sensors for the shape reconstruction process gained attention because of their independence on line-of-sight, high sensitivity, and small size. As a result of these advantages, they are widely used for reconstructing the shape of robots from continuum manipulators to needles. In this approach, the position and orientation information from several EM sensors placed along the robot body is utilized to reconstruct the shape. Due to the increase in the development of soft and disposable manipulators, recently passive stretchable sensors have been explored to be used in the shape reconstruction processes due to good features like high stretchability and low cost. Alternatively, medical imaging techniques, such as fluoroscopy, ultrasound, and endoscopic camera-based are frequently used to track the tip and estimate the shape without consuming extra space and requiring typically no major hardware modifications in robots. As these approaches produce the most straightforward and direct visualization, they play a predominant role in guiding the manipulator accurately inside the anatomy to date.

The development of shape reconstruction processes for different instruments/robots is an emerging field involving different physics and engineering aspects. They have been widely investigated by different research groups to estimate the shape of medical robots such as catheters, colonoscopes, continuum or soft manipulators using integrated sensors and medical imaging techniques. Shi et al. (Shi et al., 2017) presented a paper that described the different state of the art of shape sensing technologies using FBG, EM, and intraoperative imaging technology applied to different manipulators used in minimally invasive surgery. With the advancement of soft and flexible manipulators, recently new shape reconstruction approaches have been developed to be implemented into them. Other new designs and strategies are also proposed in the literature to increase the accuracy and robustness of previously developed methods. This review provides a summary of the current state of the art of the different shape reconstruction processes using sensors such as optical, position, and passive stretchable and imaging processes like fluoroscopy, ultrasound, and endoscopic camera-based. Each process is explored with the principle of operation involved, implemented algorithms, benefits, and associated challenges.

## Methodology

**FIGURE 1:**
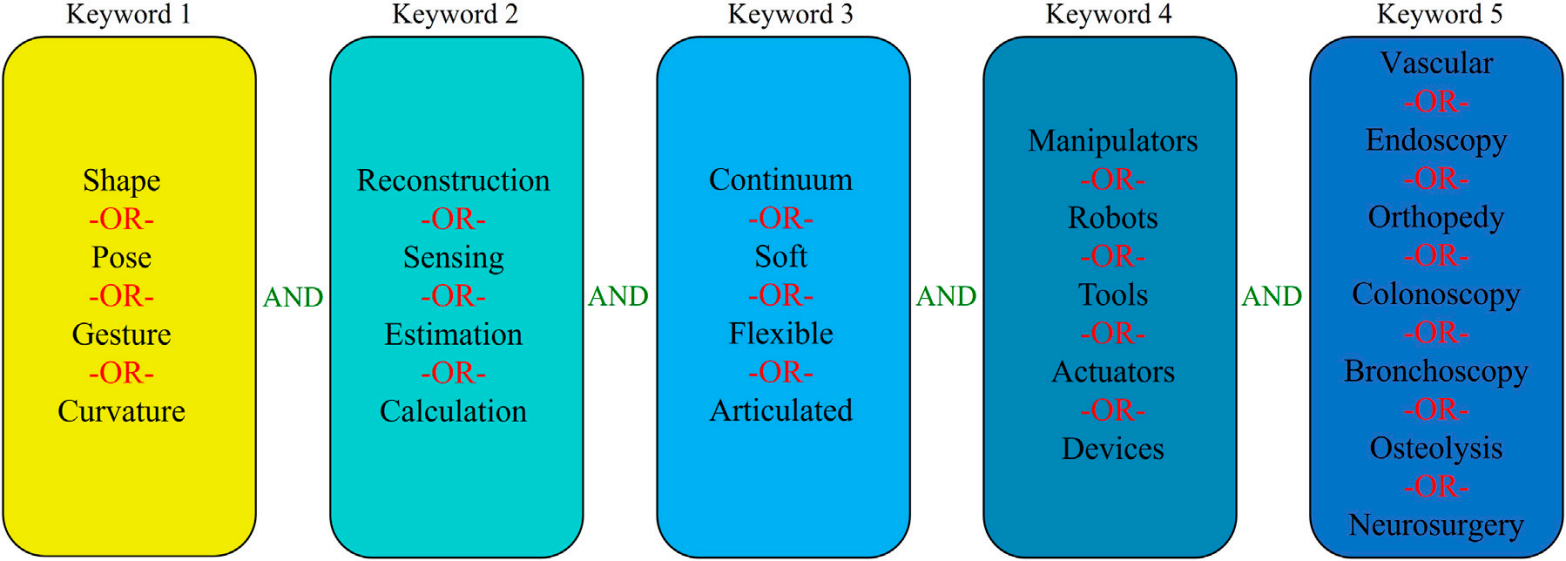
Methodology of literature research.

The field of shape reconstruction processes is broad and multidisciplinary. Therefore, to carry out a high quality survey of state of art, initially different types of shape reconstruction processes were considered based on the sensing principle. Next, the databases of Google Scholar and Scopus were used to find the most relevant articles by manual screening. Since the shape reconstruction process in interventional applications is a growing field, in this paper the search was limited to the literature published in the recent 30 years. The list of keywords was selected based on their use in interventional applications and then combined with logical operators to find the most relevant articles. Figure 1 shows the overall structure showing the combination of keywords used during the search.

The use of keywords in Google Scholar and Scopus databases yielded several articles, out of which, the duplicate findings were removed, and the remaining articles were scanned by reviewing the abstracts to discard the unrelated papers. The rest of the articles are classified into different categories based on the sensing principle, which were reviewed and presented in this work.

## Shape Reconstruction Processes

**FIGURE 2:**
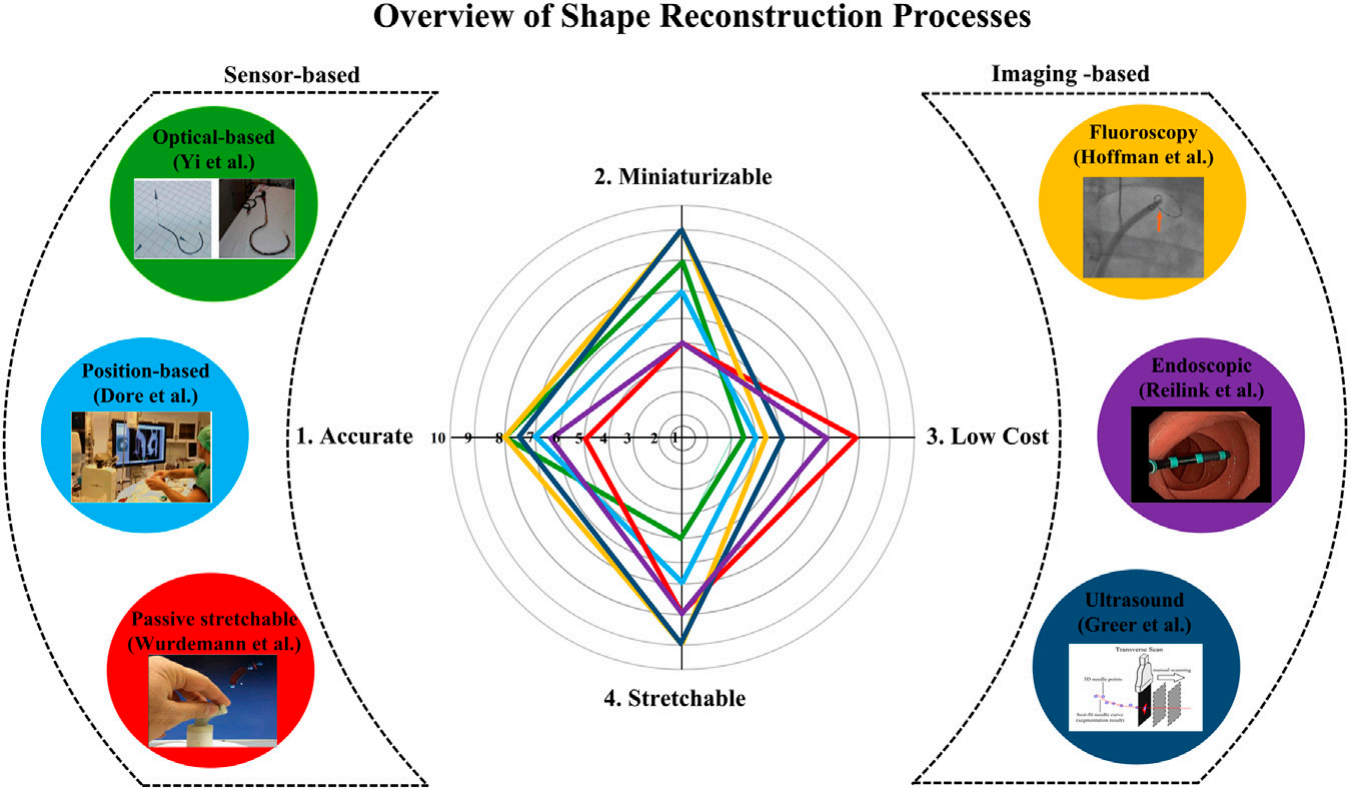
Classification of sensor-based **(Left)** and imaging-based shape reconstruction process **(Right)**, and the comparison of each technique according to the properties **(Middle)**.

Shape reconstruction processes are typically classified into two categories based on the type of technologies involved. The first one is called sensor-based, in which a series of sensors are integrated into the manipulators for reconstructing their shape. In this case, the most frequently employed sensors are optical sensors, position sensors, and passive stretchable sensors. The shape reconstruction process is performed by acquiring the strain or positional information from these sensors. The second one is called imaging-based processes, as it uses different imaging modalities such as fluoroscopy, endoscopy, and ultrasound for shape reconstruction. Unlike sensor-based processes where the position and strain information from the sensors is crucial, these technologies reconstruct the shape by processing the acquired images. Figure 2 shows the classification of the shape reconstruction processes employing sensor-based and imaging-based technologies. Based on the search process described in *Methodology*, the articles are arranged according to the above categories. Among the sensor-based processes, optical fiber techniques showed widespread exploration in the literature; hence they have been discussed first. Next, the position sensor-based processes are discussed, and finally, the passive sensor-based processes are reviewed as they are the least explored techniques compared to the others. The same procedure has been followed for image-based shape reconstruction processes when selecting the sequence which follows this order: fluoroscopy, ultrasound, and endoscopic camera-based.

Based on the above classification, this paper discusses the applicability of each sensing methodology in terms of the size of the instruments/robots used (miniaturization), cost, shape reconstruction error, and stretchability challenges. According to the author’s inference from the state of the art, a comparison between the shape reconstruction processes is carried out where each methodology is rated from 1 to 10 for each property summarized in Figure 2. This figure is based on the opinion of the authors and shows a broad overview of the various shape reconstruction processes, and their advantages and limitations.

From the comparison (Figure 2), it can be stated that when cost and stretchability are primary criteria, shape reconstruction using passive stretchable sensors and endoscopic camera-based processes can be adapted to the applications. However, they may not be implemented in small-sized robots and could increase the reconstruction error due to 1) obstruction or occlusion in front of the camera for endoscopic camera-based techniques and 2) hysteresis involved in case of passive stretchable sensor-based processes. If accuracy and miniaturization are the main criteria, then imaging-based processes such as fluoroscopy and ultrasound, and FBG sensor-based methods can be applied. However, issues like stretchability and fabrication challenges in the case of FBG and the use of bulky instruments in imaging methods should be considered. The stretchability issue of FBG can also be addressed by imaging modalities as they don’t involve any sensor in the robot body. However, high radiation dosage and low signal-to-noise ratio of imaging-based processes create challenges in some applications. From the figure, it can also be observed that the position-based shape reconstruction shows moderate accuracy, and cost and can be implemented to small flexible robots too. However, as the position-based methods are dealing with electromagnetic fields, EM interferences should be considered. In the following sections, these properties of each shape reconstruction process are described in detail.

## Sensor-Based Shape Reconstruction Processes

Sensor-based shape reconstruction processes are divided into three categories which are fiber optic-based, position-based and passive stretchable sensors-based. Each category will be discussed in detail in the following sections.

### Fiber Optic-Based Shape Reconstruction Processes

The first sensor-based shape reconstruction is fiber optic sensors-based. These sensors consist of a light source, a carrying medium that is sensitive to environmental measurands, and a receiver (Fidanboylu and Efendioglu, 2009) which detects the modulations of the emitted light in terms of intensity, wavelength, phase, polarization (Krohn et al., 2014). Thanks to their miniature size, immunity to electromagnetic interference, and flexibility, they are used to develop various types of sensors from strain to chemical measurement (Udd, 1995).

This section presents sensing methods that have the potential to reconstruct the shape of the continuum robots through strain information from fiber optic sensors. After a brief discussion of intensity-modulated, phase-modulated, and scattering-based methods, Fiber Bragg Grating (FBG), an extensively studied technique of wavelength-modulated method, is presented.

In intensity-modulated methods, an optical fiber is equipped with a light source and a photodetector. When a measurand causes deformation in the sensor, the intensity of the detected light modulates (Santos and Farahi, 2014) due to reflection, transmission loss, micro/macrobending (Anwar Zawawi et al., 2013). Zhao et al. demonstrated curvature sensing for orthotic systems (Zhao et al., 2016) which consisted of U-shaped fibers for sensing. One side of each U-shaped fiber was etched to reduce internal reflection, resulting in loss of light while traveling. As one side is etched, the amount of light dissipation was affected during bending, which was measured via a photodetector and then correlated to the curvature.

Similarly, Searle et al. showed the curvature sensing of a flexible manipulator (Searle et al., 2013), which consisted of fiber optic cables and reflective surfaces. The distance between the reflective surfaces and fiber ends varied with the bending of the manipulator, which caused a change in reflected light and allowed correlation with the bending measurement. Sareh et al. presented pose sensing of a soft actuator (Sareh et al., 2015) in which three stretch sensors were sewn into the braided sleeve layer of the actuator. When the actuator bends, the measurable light varies due to macrobend loss allowing to detect the bending angle. To et al. presented a soft sensor that can measure the curvature by coating the stretchable waveguide with a non-stretchable and reflective metal layer (To et al., 2015). When the sensor undergoes a deformation, micro-cracks within the reflective layer cause the loss in intensity of the light.

Phase-modulated fiber optic sensors work on detecting the phase change of the light. Typically, a modulation in phase because of a measurand is detected interferometrically by comparing an isolated reference phase (Krohn et al., 2014) using Fabry-Perot, Mach-Zehnder, Michelson, or Sagnac method (Lee et al., 2012). Compared to intensity-modulated, it shows higher sensitivity and accuracy but requires more complex interrogation and data processing techniques (Roriz et al., 2012). Zhou et al. presented a curvature sensor (Zhou et al., 2020) that included a dual-core photonic crystal fiber (PCF) spliced between two single-mode fibers. When the PCF is bent, phases of the propagating light at the PCF cores differ because of the inherent reflective index variations and changes in the optical path. The phase difference was used for curvature detection using Mach-Zehnder interferometry.

Scattering-based fiber optic sensors catch the scattering of propagating light through an optical fiber and the intensity of the scattered light is mapped using a reflectometer (Di Sante, 2015) by using Rayleigh, Brillouin, or Raman mechanism. Rayleigh scattering occurs when propagating light collides with inhomogeneities in the fiber optic core (i.e., structural variations, impurities (Bao et al., 2019)). The inhomogeneities act as a scattering center, reflecting light in nearly all directions with negligible energy loss. Moreover, this elastic scattering does not change the frequency (Bao et al., 2019). Unlike Rayleigh, Brillouin and Raman scattering mechanisms are inelastic, and there is a change of energy, resulting in frequency change (Krohn et al., 2014). Galloway et al. presented 3D shape sensing of a soft actuator (Galloway et al., 2019) in which a multicore fiber with one central and three outer cores that helix around the center core was placed into it. Under deformation, the outer cores were subjected to tension/compression while the center core remained neutral, changing backscattered signals. Then, the scattering was detected using optical frequency domain reflectometry. Since the input light was split between a reference path and a measurement path which was combined with backscattered signals, the shape of the actuator was reconstructed by comparing the reference and measurement signals.

The intensity and phase-modulated methods mainly focus on single bend curvature sensing. Therefore, they can be adapted to medical robots with single bend curvature. However, in continuum robots, multi-bending with accompanying torsion is common. By using the scattering-based method, curvature and torsion sensing were demonstrated (Galloway et al., 2019). However, further investigation is required to detect multi-bending mode.

On the other hand, wavelength-modulated methods detect wavelength shift, which is independent of the light source intensity (Krohn et al., 2014). The following section summarizes the FBG methods used for 2D/3D shape reconstruction of continuum manipulators with single and multi-bending modes.

FBG is a type of intrinsic sensor (Urban et al., 2010) which is deployed by changing the refractive index of the core of the optical fiber. Chronologically, the fabrication techniques of the FBGs are photosensitivity (Hill et al., 1978), holography (Meltz et al., 1989), phase masking (Hill et al., 1993), and femtosecond laser (Martinez et al., 2004). When an incident spectrum of light propagates through the FBG, a narrow wavelength of light is reflected while the rest is transmitted. The reflected wavelength is called Bragg wavelength which has cross-sensitivity to strain and temperature. Thus, when an FBG is subjected to them, the reflected Bragg wavelength shifts proportionally. The FBG response is characterized by measuring the shift using an interrogation system. Moreover, the Bragg wavelength can be adjusted in the design stage through changing effective refractive index (Huang et al., 2008), cladding/core indexes (Oliveira et al., 2018), and grating period (Patrick et al., 1996). This adjustment is useful for multiplexing which allows for inscribing many independent FBGs along the same fiber while avoiding the overlapped Bragg wavelengths. It allows for monitoring different variables (Mizutani and Groves, 2011), (Pan et al., 2015) or distributed/quasi-distributed measurement of a single variable (Igawa et al., 2008), (Kanellos et al., 2010) using one fiber with FBGs.

In medical applications, FBGs are proposed for the shape reconstruction of continuum instruments since it is crucial to know the instrument shape to reach a target area by avoiding anatomical obstacles and sensitive tissues. Miniature size, work over a long distance, immunity to the electromagnetic fields, chemical inertness, high sensitivity and repeatability, and fast response features (Werneck et al., 2013) make the FBG technology a prominent candidate to be used for shape reconstruction of continuum robots in the medical field.

The shape of continuum robots changes through curvature, torsion, extension/contraction, and their combinations along their body (Walker, 2013). When the shape of a continuum body changes, the induced strain at the FBG causes the wavelength shift, then the shape is reconstructed by characterizing this shift using the following steps typically: 1) strain on each fiber is calculated through the wavelength shift; 2) using the strain, curvature and torsion are computed; 3) missing curvature and torsion are obtained through interpolation; 4) shape is reconstructed using obtained data (Jäckle et al., 2019). The FBG-based shape reconstruction is applied to various continuum structures, such as tendon-driven manipulators (Wei et al., 2017), (Roesthuis et al., 2013), (Liu et al., 2015a) (Sefati et al., 2019), pre-curved robots (Xu et al., 2016), soft actuators (Wang et al., 2016; He et al., 2018; He et al., 2019; Wang et al., 2020), catheters (Khan et al., 2019), (Shi et al., 2014b), and needles (Park et al., 2010), (Roesthuis et al., 2014). Moreover, it was proposed as an external sensor to be inserted into different continuum instruments, as summarized in Table 1.

**TABLE 1:** Summary of FBG based Shape Reconstruction Processes.

Application	Author	Design Parameter	Method	Testing/medical Scenario	Evaluation
Tendon-driven manipulator	Liu et al. Liu et al. (2015a), Liu et al. (2015b)	- 2 fibers with 3 FBGs each	**(i)** The manipulator under the assumption of constant curvature Liu et al. (2015a)	Osteolysis	**(i)** The average curvature error in 2D: 3.14% Liu et al. (2015a)
		- The fibers are reinforced with Nitinol wires and embedded into the wall of the manipulator	**(ii)** The manipulator under the assumption of non-constant curvature Liu et al. (2015b)		**(ii)** The maximum tip tracking error in 2D: 0.4 mm in free bending, 0.93 mm in bending with an obstacle Liu et al. (2015b)
Tendon-driven manipulator	Sefati et al. Sefati et al. (2019)	- 3 fibers with 3 FBGs each	The manipulator shape is reconstructed by using	Orthopedy	The maximum tip position error in 2D
		- The fibers are attached around a Nitinol wire in a triangular configuration	**(i)** traditional model-dependent approach,		**(i)** 3.63 mm (model-dependent),
		- The wire with fibers is embedded into the wall of the tendon-driven manipulator	**(ii)** proposed data-driven approach		**(ii)** 0.62 mm (data-driven)
Tendon-driven manipulator	Roesthuis et al. Roesthuis et al. (2013)	- 3 fibers with 4 FBGs each	The manipulator under the assumption of constant curvature	Minimally invasive surgery	The maximum tip trajectory tracking error for open loop (closed loop)
		- The fibers are attached around a Nitinol wire in a triangular configuration			-Circle trajectory (2D): 10.47 mm (0.67 mm)
		- The wire with fibers is inserted into the hollow backbone of the manipulator			-Square trajectory (2D): 11.7 mm (1.71 mm) -Helix trajectory (3D): 14 mm (0.87 mm)
Tendon-driven manipulator	Wei et al. Wei et al. (2017)	- 1 fiber with 8 FBGs	A new model is presented for the helical arrangement to detect curvature and torsion measurement	Natural orifice translumenal endoscopic surgery	The maximum tip position error in 2D: Less than 0.1 mm for both *x* and *y* axes
		- The fibers are supported with a Nitinol wire			
		- The fiber with wire is mounted on a surface of the manipulator in a dual helical configuration			
Pre-curved robot	Xu et al. Xu et al. (2016)	- 3 fibers with 1 FBG each	In addition to shape sensing considering curvature and torsion, the lateral tip force is calculated	Neurosurgery	The maximum curvature and torsion error
		- The fibers are wrapped helically around a pre-curved Nitinol wire			-In *x*-axis: 0.0753 rad/m
					-In *y*-axis: 0.1115 rad/m
					-In *z*-axis: 0.0616 rad/m
					The maximum force sensing error: 7.07 gf
Soft robot	He et al. He et al. (2018), He et al. (2019)	- 1 fiber with 2 FBGs	Reconstruction from curvature by using differential geometry	General purpose soft surgical robot	The maximum sensitivity: 50.15 pm/m^−1^ at 30 m^−1^ with fluctuating interval of 4.67%
		- The fiber is embedded with an offset from the neutral bending line of the pneumatically driven soft actuator			
Catheter	Khan et al. Khan et al. (2019)	- 4 multicore fibers, each fiber with 4 cores, each core with 6 FBGs	Curvature and torsion are calculated using the Frenet-Serret equation	General purpose catheter	The maximum reconstruction error in 3D: 1.05 mm
		- The fibers are placed into the catheter			
Catheter	Shi et al. Shi et al. (2014b)	- 3 fibers with 8 FBGs each	Curvature and torsion are calculated using the Frenet-Serret equation	Transcatheter aortic valve implantation	Not reported
		- The fibers are assembled in a triangular configuration			
		- The assembled fibers are attached on the catheter			
Needle	Park et al. Park et al. (2010)	- 3 fibers with 2 FBGs each	The beam theory was applied to estimate the deflection profile of the needle	MRI-guided interventions	The maximum RMS error of the tip deflection in 2D (in water bath): 0.38 mm in ±15 mm deflection
		- The fibers are placed into the needle in a triangular configuration			
Needle	Roesthuis et al. Roesthuis et al. (2014)	- 3 fibers with 4 FBGs each	A kinematic-based (i.e., circle segment method that considers constant curvature approximation) and mechanics-based models are developed and applied	General purpose needle	The maximum reconstruction error in free space (gelatin phantom)
		- Fibers are placed into the needle			-In-plane with single bend: 0.20 mm (0.57 mm)
					-In-plane with double bend: 0.51 mm (0.53 mm)
					-Out-of-plane:1.66 mm (0.74 mm)
External Tool	Ryu and Dupont Ryu and Dupont (2014)	- 3 fibers (1 fiber with 1 FBG, 2 fibers without FBG)	The tool under the assumption of constant curvature	To reconstruct the shape of continuum robots	The mean tip position error: 0.84 mm
		- The fibers mounted on the surface of the polymer tube in a triangular configuration			The mean tip orientation error: 1.21°
External Tool	Lunwei et al. Lunwei et al. (2004)	- 4 fibers with 5 FBGs each	Reconstruction in 2D (from curvature) and 3D (from curvature and torsion) by using differential geometry	To reconstruct the shape of colonoscopes	The minimum error: 4.1 mm
	Yi et al. Yi et al. (2007)	- The fibers are placed around the shape memory alloy wire			
External Tool	Jäckle et al. Jäckle et al. (2019)	- 1 multicore fiber that includes 1 center and six equally distributed cores, each core with 38 FBGs	Frenet-Serret, parallel transport, and circle segment methods are tested. The circle segment method in Roesthuis et al. (2014) is selected due to fast convergence and low error	To reconstruct the shape of flexible instruments for endovascular navigation	The maximum error
					(i) 7.53 mm (in free space),
					(ii) 2.11 mm (in endovascular scenario)

**FIGURE 3:**
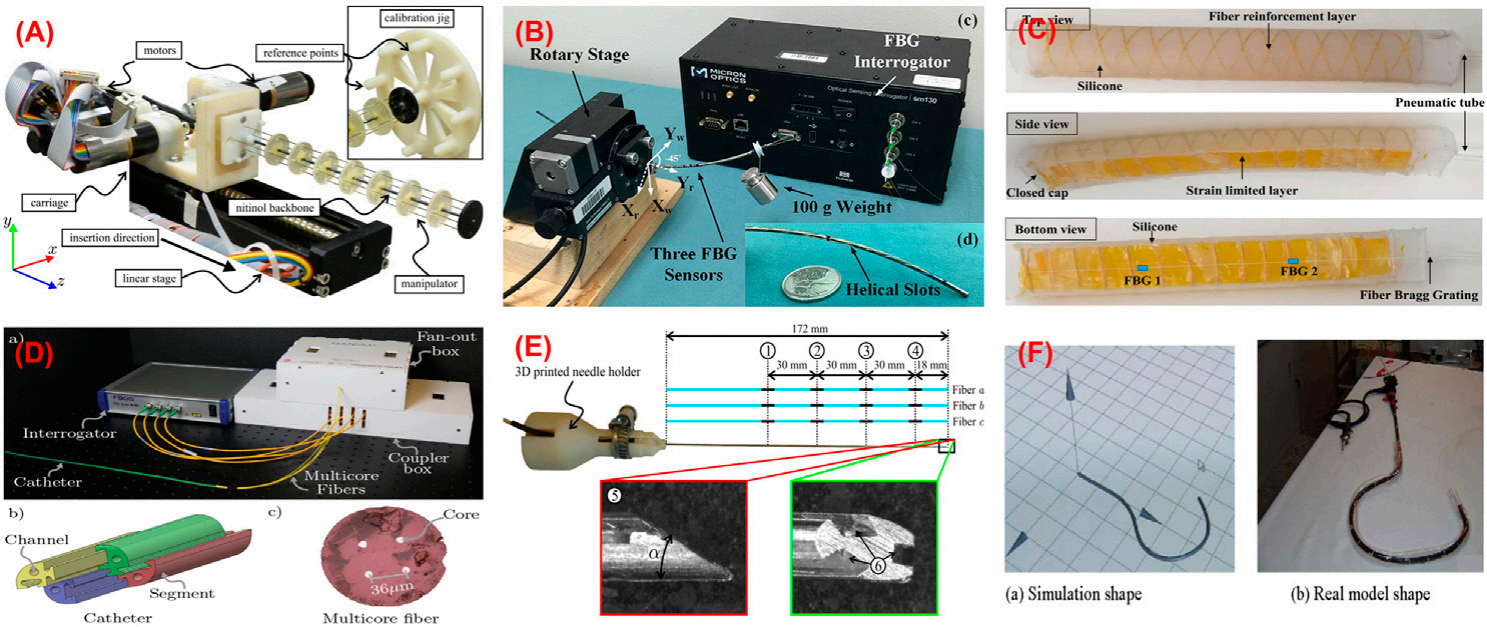
Shape sensing using FBG. **(A)** Tenon driven manipulator (Roesthuis et al., 2013); **(B)** pre-curved nitinol tube (Xu et al., 2016); **(C)** soft manipulator (He et al., 2018); **(D)** catheter (Khan et al., 2019); **(E)** needle (Roesthuis et al., 2014); **(F)** colonoscope (Yi et al., 2007).

Regarding tendon-driven manipulators, Liu et al. presented FBG-based shape reconstruction of a large curvature manipulator (Liu et al., 2015a). The maximum curvature of the manipulator was much higher than the maximum bending strain of the fiber; thus, each fiber with three FBGs was coupled with Nitinol wires as a supporting substrate in a triangular configuration, aiming to reduce the bending strain on the fibers. Experimental tests were conducted in-plane under constant curvature assumption, and the average curvature error of 3.14% was reported. Then, the study was extended for non-constant curvature detection in (Liu et al., 2015b). Experiments were evaluated in free bending and bending with an obstacle case which produced maximum distal tip tracking error of 0.4 and 0.93 mm, respectively. It allows detecting large deflection. However, this deals with a complex fabrication method (Sefati et al., 2017). Sefati et al. presented a method for fabricating a large deflection FBG sensor by embedding one FBG array and two NiTi wires inside a polycarbonate tube (Sefati et al., 2016). This sensor was used for shape reconstruction and controlling a continuum manipulator (Sefati et al., 2018). In (Sefati et al., 2019), the shape of the similar manipulator was reconstructed by using model-dependent and data-driven approaches. It was reported that the data-driven approach uses all FBG’s data simultaneously and does not rely on geometrical assumptions as in the model-dependent approach, resulting in reducing reconstruction error, especially in large deflection. The maximum absolute error of 3.63 and 0.62 mm were reported for model-dependent and data-driven approaches, respectively. The same group proposed another data-driven learning model (Sefati et al., 2021) for shape reconstruction of a similar manipulator. This method incorporated three supervised machine learning algorithms, which were trained on the collected data to map measurements from FBG to the distal-end position. This measurement was further used in a data-driven optimization-based shape reconstruction process to reconstruct the manipulator shape. When the data-driven approach was compared with the model-dependent approach, it produced a maximum distal end position error of 1.22 mm for data-driven and 3.19 mm for model-based which showed a good performance of the data-driven process. Roesthuis et al. proposed a 3D shape sensing of a tendon-driven manipulator (Figure 3A) for closed-loop control (Roesthuis et al., 2013). In this case, a Nitinol wire of 1 mm in diameter and 160 mm in length with an integrated array of FBGs serves as the sensor for shape reconstruction. The Nitinol wire comprised three optical fibers and each fiber consisted of four FBG nodes. Then, the wire with fibers was inserted into the backbone of the manipulator to estimate its curvature by measuring axial strain on FBG sensors. After that, the shape was reconstructed using the curvature information which was further used to steer the manipulator tip. It was demonstrated that tip tracking error was significantly reduced thanks to reconstruction feedback from FBGs. This study was also extended (Roesthuis and Misra, 2016) to find interaction force while performing shape sensing simultaneously using rigid-link modeling. The 3D shape generated from this process was used as feedback for control. Experimental results showed a maximum trajectory error of 1.37 mm when the manipulator was steered along a straight path. In addition to the straight fiber configurations in (Roesthuis et al., 2013), (Liu et al., 2015b), (Sefati et al., 2019), Wei et al. proposed a helical configuration of a single fiber with eight FBGs for a tendon-driven flexible robot (Wei et al., 2017). The fiber was supported with Nitinol wire. The dual-helical (i.e., clockwise and counterclockwise) configuration of the fiber had the potential of torsion and curvature measurements as well as temperature compensation. The FBGs along the fiber was placed with axisymmetry according to the neutral axis of the robot. This placement aimed to produce the same wavelength shift but in the opposite strain sign at the two axisymmetric FBGs, which can also be used for temperature compensation. The torsion and curvature tests were conducted separately. For the torsion test, a fiber that has 4 FBGs with Nitinol wire was bounded around a 20 mm diameter silicone rubber shaft. Results showed that wavelength shifts linearly when one end of the shaft was rotated 360° while the other end was fixed. To demonstrate constant curvature in-plane bending, a fiber that has 8 FBGs with Nitinol wire was placed onto a 25 mm diameter and 110 mm long 3D printed flexible robot. The curvature experiments were compared with camera images which resulted smaller than 0.1 mm in-plane bending error.

Regarding pre-curved robots, Xu et al. demonstrated shape and force sensing of a pre-curved Nitinol tube (Xu et al., 2016). Three fibers with one FBG each were helically wrapped around a tube (Figure 3B). A nonlinear force-curvature-strain model was developed to measure curvature, torsion, and force simultaneously based on strain changes of the FBGs. In addition to shape and force sensing, the effect of FBG length was investigated. 2 FBG sensors (i.e., 1 and 5 mm) were tested in terms of bending capacity and noise. It was reported that while FBG with 1 mm in length allows larger bending than 5 mm one, the latter shows a better resolution. To obtain larger bending, FBGs with 1 mm in length were used during shape and force sensing of pre-curved tube experiments. After calibration, a series of weights (up to 150 g) was added to the distal end of the tube. Under maximum loading, the error for curvature measurement was reported as 2–4% of the total range measured.

With the increasing application of soft robots, FBG-based shape reconstruction processes are applied to soft structures (He et al., 2018), (He et al., 2019), (Hou et al., 2021). He et al. presented the shape sensing of a pneumatic soft bending actuator (Figure 3C) (He et al., 2018). A fiber with two FBGs was placed on the bottom surface of the 180 mm in length actuator. The maximum accuracy was reported as 50.15 pm/m^−1^ at 30 m^−1^ curvature, and the fluctuating interval is 4.67% in bending (He et al., 2019). In another approach, Hou et al. proposed a new FBG-based pose reconstruction method using an improved piecewise constant curvature model for a soft manipulator of length 154 mm (Hou et al., 2021). In this case, a helical design of FBG considering the axial elongation/compression of the manipulator was proposed, which has the potential to measure large deformations. The experimental results showed an interesting accuracy with a maximum error of the end effector coordinates 0.76 mm when compared with the ground truth coordinates given by vision. However, there is no available torsion information.

These processes for catheters were also widely investigated (Shi et al., 2014b), (Zhang et al., 2020) (Al-ahmad et al., 2020). Khan et al. presented the 3D reconstruction of a catheter (Figure 3D) using four multicore fibers (each fiber with four cores and each core with six FBGs) into a 118 mm long catheter (Khan et al., 2019). Although one multicore fiber with three or more cores is enough for 3D shape reconstruction, they used a redundant multicore fiber to increase reliability against sensor failure. Experiments were conducted in various curvature and torsion conditions, reporting the maximum reconstruction error of 1.05 mm. Moreover, Shi et al. proposed the reconstruction of a 1,000 mm long catheter with three optical fibers with eight FBGs each (Shi et al., 2014b) assembled in a triangular configuration. Although the result of a single bend mode was presented, the accuracy of the sensor was not reported. Recently, Fei Qi also presented reconstruction process for a catheter using the curvature information from the FBG sensor in a discrete interpolation fitting method (Qi et al., 2021). The information was used in a control strategy to improve the bending accuracy of the robot. Results showed that the curvature error and direction angle errors are 1.42 and 10.3%, respectively.

In the case of needles, Park et al. presented shape reconstruction of a 150 mm long MRI-compatible biopsy needle (Park et al., 2010). Three fibers with two FBGs each were placed into the needle shaft in a triangular configuration. It was calibrated by applying in-plane loads and changing temperature without strain which was used for shape reconstruction. The maximum RMS error of the tip deflection was reported as 0.38 mm in the range of ± 15 mm while the temperature was compensated. On the other hand, Roesthuis et al. presented a 2D and 3D shape sensing of a 172 mm long Nitinol needle (Roesthuis et al., 2014) by placing three fibers with four FBGs in a triangular configuration (Figure 3E). The shape was reconstructed from curvature and torsion through a beam theory-based model. The experiments were conducted in free space and gelatin phantom in which the maximum reconstruction errors in free space (gelatin phantom) were presented as 0.2 (0.57) mm for in-plane with a single bend, 0.51 (0.53) mm for in-plane with a double bend, and 1.66 (0.74) mm for out-of-plane. Kim et al. proposed another method based on elastic rod theory and Lie-group-theoretic approach to reconstruct the shape of a needle used in MIP (Kim et al., 2017a). This approach used the information from 3 embedded FBG sensors to reconstruct the shape. Two tests were performed where the needle was inserted into a single layer and a double layer homogeneous phantoms and the reconstructed shape was compared with the shape produced from image analysis. The mean tip deflection error was estimated as 0.2 ± 0.12 mm for the single layer and 0.47 ± 0.17 mm for the double layer phantom, respectively. Later, this study was extended to test the shape sensing capability of the needle (Kim et al., 2017b) when inserted into an inhomogeneous phantom consists of soft gel and meat, which produced a maximum error in the tip deflection as 0.38 ± 0.27 mm.

Alternatively, the FBGs are also used to develop external tools to be inserted into channels of flexible instruments. This approach separates the sensors from the robot body while using minimum usage of structure lumen and providing the potential of inexpensive fabrication and maintenance (Ryu and Dupont, 2014). After the 3D shape sensing concept of a colonoscope was suggested in (Lunwei et al., 2004), Yi et al. presented the details in (Yi et al., 2007) as follows. A shape memory alloy of 0.76 mm in diameter was equipped with four fibers with five FBGs each. The sensor was used to reconstruct the shape of the colonoscope inserted into its biopsy channel. The shape of the colonoscope was reconstructed based on differential geometry, considering curvature and torsion in the ambient temperature. After a calibration matrix was obtained considering sensor packing error, the shape of the sensor was reconstructed for in-plane bending and spatial conditions (Figure 3F), reporting the minimum error of 4.1 mm. As a different application, Ryu and Dupont developed a sensing tube that includes three surface-mounted fibers in a triangular configuration (Ryu and Dupont, 2014). A 1.4 mm in outer diameter tube tends to use the minimum amount of lumen, not to affect the curvature of the robot which is inserted into. Moreover, it allows integrating various tools as demonstrated with micro forceps. The sensing tube allows large curvature and strain reduction between fiber and compliant material. However, it reduces the strain transfer from compliant polymer to stiff fiber. To address that, a mechanics-based strain transfer model was derived and validated through simulation and experiments. A low-cost prototype with one fiber with a single FBG and two fibers without FBG was tested in-plane bending. A robot of constant curvature and 80 mm in length was assumed according to the intracardiac scenario. While tip position error was reported as 0.84 mm, tip orientation error was given as 1.21°. On the other hand, Jäckle et al. used a multicore fiber for shape sensing of flexible instruments for endovascular navigation purposes (Jäckle et al., 2019). A 380 mm in length multicore fiber has one central core and equally distributed six outer cores, each containing 38 FBGs. For 380 mm sensor length, experiments in free space resulted in an average error of 0.35–1.15 mm and maximal error of 0.75–7.53 mm. In the scenario with endovascular reconstruction, they obtained an average error of 1.13 mm and a maximal error of 2.11 mm.

All in all, the FBG-based strategy is a promising candidate for the 2D/3D shape reconstruction of continuum robots. The small diameter of fibers allows shape reconstruction of structures in 1 mm or even smaller diameters. The average shape estimation error reported in the literature varied from 0.38 to 7.53 mm depending on the applied instrument and tested configuration. In the case of shape reconstruction of needles, this method has the capability to estimate the shape achieving submillimeter accuracy, i.e., error <0.5% of the length (Roesthuis et al., 2014). While for catheter and flexible instruments, the reconstruction approach is able to achieve error less than 0.9% for simple C-curve (Jäckle et al., 2019), (Khan et al., 2019), and it can reach up to 2% for more complex configurations like S-curve (Jäckle et al., 2019). Though this method produces many advantages such as accuracy, miniaturization, and high sensitivity, it has some limitations such as cross-sensitivity and limited stretchability. Since FBGs have sensitivity to temperature, various approaches were suggested to eliminate the temperature effect to increase the shape reconstruction accuracy. To mention the stretchability challenge, especially for detecting large curvature, a reinforcement element to be attached to fibers such as Nitinol wire was used to decrease the amount of induced strain on the fibers. On the other hand, various design parameters, such as the fiber core number (i.e., single-core or multicore), the optical fiber number (i.e., one, two, three, and four fibers), and the fiber configurations (i.e., straight or helical) were discussed. Among the parameters, it can be seen that three optical fibers in a triangular configuration are commonly used. This configuration allows measuring curvature and torsion while eliminating the common noise and temperature effects. Although this configuration is enough for 3D shape sensing, it is reported that a redundant number of multicore fibers was used to increase shape reconstruction accuracy.

### Position-Based Sensor Shape Reconstruction Processes

The second category of sensor-based reconstruction processes is position-based. Typical position sensors are the kind of sensors that can measure the relative position and orientation from a fixed or reference point. Examples of these sensors are electromagnetic (EM) and permanent magnet trackers, inertial measurement units (IMU), etc. These sensors are very widely used (Franz et al., 2014) in MIP to track the position of the tool or robots involved due to their advantages, such as real-time tracking, freedom from the line of sight, and accuracy. Recently, they are also gaining attention for reconstructing the shape of robots due to these advantages. In this section, the use of EM and permanent magnet trackers in the shape reconstruction process is discussed. The working principle of EM trackers is based on mutual induction in which an EM field generator generates a known electromagnetic field to determine the pose information of the sensors within its workspace. On the other hand, the permanent magnets are tracked by measuring the magnetic field change due to its movement using a sensor system. Position-based shape reconstruction produces certain advantages over FBG shape reconstruction, such as freedom from line-of-sight and allowing for reconstructing the shape of a highly flexible robot.

#### Electromagnetic Tracking-Based Shape Reconstruction Processes

Electromagnetic tracking is widely used in different medical applications starting from image-guided interventional surgery (Xiao et al., 2018) (Cleary et al., 2005), more recently, to medical device navigation (Linte et al., 2012), (Aufdenblatten and Altermatt, 2008), thanks to their miniaturization, precision, easy to set up installation, and freedom from the line-of-sight. Consequently, electromagnetic tracking is a promising method for tracking and localizing the devices in clinical applications such as bronchoscopy (Leong et al., 2012), endoscopy (Fried et al., 1997), knee arthroplast (Lionberger et al., 2008). In addition to these applications, EM sensors are also used for shape reconstruction of medical devices by placing multiple EM sensors along the length of the device. The EM shape reconstruction process depends upon the location and orientation of the sensors attached to the device without requiring curvature information. This section provides the overall idea of shape reconstruction processes of manipulators used in interventional procedures with Table 2 providing the summary of the different existing processes.

**TABLE 2:** Summary of position-based shape reconstruction processes.

Application	Author	Design Parameter	Method	Testing/medical Scenario	Evaluation
Articulated snake robot	Tully et al. Tully et al. (2011), Tully and Choest (2016)	One 5 degree of freedom EM sensor at the tip of an articulated snake robot	Fusing measurements from magnetic tracker and information from kinematic model	In a benchtop experiment as well as with epicardial surface of a porcine subject	Maximum shape estimation error for bench top experiment 10.53 mm
Wire riven robot	Song et al. Song et al. (2015b)	3 six DoF EM sensors placed along the length of a wire driven robot	A reconstruction algorithm based on three order Beizer curve using the EM information from sensor and robot length	Free space	Mean reconstruction error found as 1.7 mm
Flexible manipulator	Ma et al. Ma et al. (2018)	Three EM sensors attached on base, middle segment, and end effector of a flexible manipulator	EM sensors data used with third order Bezier curve and a quadratic curve fitting	Free space	Mean shape reconstruction error was 1.9 mm
Continuum manipulator	Guo et al. Guo et al. (2019)	Integrating small cylindrical permanent magnet and three axis magnetic sensors into a continuum robot	Implementation of a quadratic Beizier curve using the positional information from detection system	Free space	Maximum and minimum error of the terminal position found as 9.339 and 0.227 mm
Continuum surgical robot	Zhang et al. Zhang et al. (2017)	A small permanent magnet at distal end of a wire driven continuum surgical robot	Information from sensor array used in a three order Bezier curve	Experimental platform consists of the robot and some obstacles made from Lego	Mean position error estimated as 1.1 ± 0.5 mm
Continuum Tubular Robot	Wang et al. Wang et al. (2017)	Two small permanent magnets at the distal end of each tube of the robot	A three order Bezier curve using the information from the sensory system	Free space	Mean shape detection error was 1.38 mm
Catheter	Tran et al. Tran et al. (2017)	Five DoF EM sensors in the catheter	A probabilistic framework which merges EM measurements with dense 2D information extracted from fluoroscopic images	Validation in a physical based simulation environment	Median RMS shape sensing error was 3.7 mm
Catheter	Dore et al. Dore et al. (2012)	Seven EM sensors across the length	Combining *in situ* real-time EM tracking data of catheter with physical based catheter modelling	Inside a 2D silicone phantom of aorta	Average shape estimation error 2.1 mm

Recently, various strategies have been proposed for shape reconstruction by fusing EM tracking data and the kinematic model of the manipulators. For example, Tully et al. proposed a filtering method that estimated the shape of a 300 mm long highly articulated snake robot (Tully et al., 2011), (Tully and Choset, 2016). The non-stochastic filtering algorithm used a custom extended Kalman filter, which combined the pose data from an EM sensor present at the tip with the kinematic model of the robot to reconstruct the shape. Initially, a benchtop experiment was performed, in which the estimated shapes were compared with the true shapes of the robot generated from post-processing data producing a maximum error of 10.53 mm for a 300 mm long manipulator. In the second experiment, the shape of the robot was estimated by navigating the robot along the epicardial surface of a porcine heart. Though the method showed promising results for benchtop experiments, it was unable to provide qualitative data due to the unavailability of ground truth in animal experiments.

**FIGURE 4:**
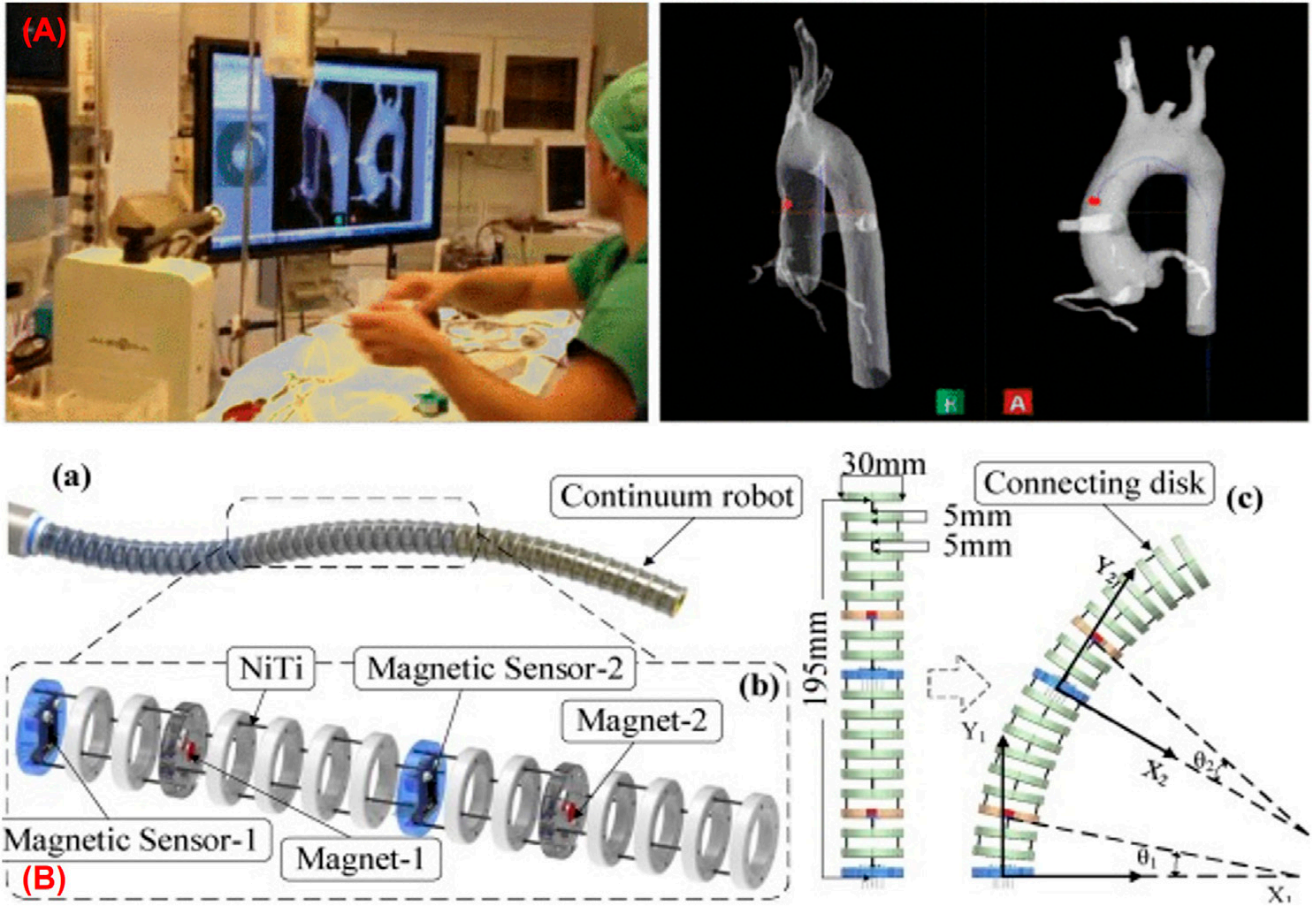
**(A)** Catheter navigation using EM sensor (Dore et al., 2012) **(B)** continuum robot shape estimation using permanent magnets (Guo et al., 2019).

In another approach, Dore et al. (Dore et al., 2012) presented catheter navigation and shape reconstruction approach by combining *in-situ* real-time EM tracking data with physical-based catheter modelling and simulation on real-time insertion length measures (Figure 4A). The catheter was comprised of seven sensors (one 6 DoF sensor at the tip and six 5 DoF EM sensors are installed at 125 mm) across its length. A probabilistic framework using Kalman Filter was employed to fuse the information from the catheter motion algorithm and the electromagnetic tracking data for estimating the shape of the catheter. The approach was validated in *in-vitro* experiments by inserting the catheter inside a 2D silicone phantom of the aorta. The average shape error was estimated as 2.1 mm compared to the ground truth resulted from the camera. Ryu et al. proposed a method to reconstruct the shape and evaluate the contact force of a colonoscope (Ryu et al., 2020). The positional and rotational information from two EM sensors connected to the distal and proximal end of the colonoscope with its mass and insertional length were used in a model to reconstruct the shape. Apart from the shape reconstruction process, the contact force was also estimated by employing the Cosserat-rod theory. The position error for specific node points was calculated by comparing to the simulated shape which remained in between 2 and 3 mm even if the sensing length changed from 300 to 700 mm.

The accuracy of the methods presented depends upon the model, and they may not work in an unknown payload if the models do not include the loading condition. Some models also use constant curvature assumption which is very difficult to avail practically. Song et al. presented a shape reconstruction method for flexible robots based on Bezier curves using the length of each section along with positional and directional information of the distal end of each section of the robot (Song et al., 2015a), (Song et al., 2015b). This method shows high accuracy in shape reconstruction with marginal modification to the robot, wherein no kinematic model for reconstruction is needed. This method was first applied in a single section robot of 135 mm in length by mounting a 5 DoF EM senor on the tip and then extended to the robot with two bending sections by placing three 6 DoF along its length. The method works well with an unknown payload applied at the tip of the robot. The veracity thereof was duly simulated and experimented where the mean reconstruction error was found as 1.7 mm. The proposed reconstruction approach provided acceptable results for shape estimation no matter how the robot bends in the same plane. However, as it used quadratic and cubic Bezier curves, the efficiency decreases if a section bends more than a specific angle (i.e., 90° for quadratic and 180° for cubic) or in a different plane. More recently, the same group also proposed a method to reconstruct the shape of a continuum tubular robot developed for nasopharyngeal biopsy (Wu et al., 2017). The robot was comprised of three EM sensors whose positional information was used in a third-order Bezier to reconstruct the shape in the 3D workspace. The reconstructed shape was compared with shapes drawn on the graph paper producing a mean error of 1 mm along the length.

Recently Ma et al. presented a novel real-time shape reconstruction process for 176 mm long and 15 mm in diameter flexible manipulator (Ma et al., 2018). In this case, the shape and twist information can be estimated simultaneously using three 6 DoF EM sensors. The proposed method reconstructed the shape using EM sensor information in a cubic Bezier curve, while twist information was calculated using EM sensor information with a polynomial fitting. This method can estimate the deformation of the manipulator with large bending along with twisting. A vision-based offline method was also developed to validate the EM sensing method showing a mean reconstruction error of 1.9 mm.

The above strategies are widely used in shape sensing and show many benefits with respect to other sensing modalities. However, they provide discrete pose information along the manipulator and require the adoption of an interpolation scheme for shape reconstruction. In addition to this, these methods produce limit robustness as they rely on magnetic field measurement, which is affected by the presence of ferromagnetic material. Some approaches using EM tracking information with other sensing modalities and predictive models (Tully et al., 2011), (Dore et al., 2012), (Azizian and Patel, 2011) are proposed to address these issues, but they require a concrete model which is very hard to achieve. Tran et al. introduced a probabilistic framework that merged EM measurements with dense 2D information extracted from fluoroscopic images to deliver a reliable estimation of the 3D shape of the catheter (Tran et al., 2017), (Tran et al., 2015). A physical-based simulation environment that gives ground truth catheter shape was developed to verify the performance of the approach, which produced a median RMS error of 3.7 mm.

#### Permanent Magnet-Based Shape Reconstruction Processes

Shape reconstruction processes based on EM sensors show numerous advantages such as high sensitivity and accuracy, miniaturization, and absence of line-of-sight. However, they need external tracking systems which add cost to the system (Wang et al., 2017), (Guo et al., 2019). Furthermore, these sensors require multiple leads for connection which affects the adaptive capacity of the robot (Zhang et al., 2017). To address the above drawbacks, alternative methods have been investigated to reconstruct the shape of the robot based on the detection of permanent magnets connected to it. Guo et al. proposed a sensory scheme by integrating small cylindrical permanent magnets and three-axis magnetic sensors into a 195 mm long continuum robot made from disks and NiTi core (Figure 4B) (Guo et al., 2019). The single section of the robot included two sets of the detection system in which each comprised of a permanent magnet and a three-axis magnetic sensor. When the robot moved, the change in the magnetic field due to the movement of the permanent magnet was measured by the magnetic sensor, which provided positional information. Then a quadratic Bezier curve was implemented using the positional information from the detection system to reconstruct the shape of the robot in the 2D plane. The maximum terminal position error was reported as 9.4 mm.

Alternatively, Zhang et al. proposed a method that illustrated the navigation of wire-driven continuum surgical robot based on permanent magnetic tracking (Zhang et al., 2017). The distal end of the robot consisted of a small permanent magnet that can provide 3D position and 2D orientation of the tip of the robot when interacting with a magnetic sensor array. Finally, using the positional information of magnets in a third-order Bezier curve, the shape of the robot was estimated. The proposed tip tracking and shape sensing method was verified using an experimental platform consisting of the robot and some obstacles made from Lego. Three targeted positions were tested using tip feedback control, and the mean position error for the tracking-based method was estimated as 1.1 ± 0.5 mm. Wang et al. (Wang et al., 2017) also presented a general joint position tracking and shape reconstruction of continuum tubular robot based on multi magnet tracking. Two small permanent magnets were mounted at the distal end of each tube of the robot. These magnets provide 3D position and 2D orientation information by communicating with the magnetic sensor array. The shape of the robot was reconstructed based on a third-order Bezier curve using the information from the sensory system. The method was verified by performing different experiments and comparing them to the actual values generated from the placement of the robot on a graph which produced a mean error of 1.38 mm.

Position-based shape reconstruction processes have many benefits like freedom from the line of sight, miniaturization, high sensitivity, and accuracy. Due to these advantages, they have been widely used in the shape reconstruction process of catheters, tubular, and articulated robots. Due to the miniature size, they have the ability to be implemented in small size continuum manipulators of radius approximately near to 2.4 mm (Dore et al., 2012). As sensors are placed at discrete points, they do not show the issue of stretchability and can be implemented in highly flexible manipulators. Despite many advantages, the methods are unable to provide uniform accuracy throughout the tracking area, as the accuracy depends upon the tracking volume. The issue was addressed by Reichl et al. (Reichl et al., 2013) by proposing an EM servoing method in which the EM field generator was connected to a robot arm. In this case, the EM tracking detects the sensors in a subspecific volume with acceptable accuracy, and then the robot was positioned to keep the sensors near to the center of tracking volume during movement. Another drawback of these methods is that the sensitivity and tracking accuracy is compromised due to the presence of a surrounding magnetic field and electrical equipment. Different compensation methods have been adopted to reduce these effects on the tracking accuracy. However, further improvement is needed in this field to bring these methods into clinical scenarios.

### Passive Stretchable Sensor-Based Shape Reconstruction Processes

Currently, passive stretchable sensor-based shape reconstruction processes such as resistive and capacitive sensors are gaining popularity due to many advantages compared to available shape reconstruction processes. These sensors are defined as passive as they measure different properties by changing some passive electrical quantities such as resistance and capacitance. Due to their high stretchability, they are mainly adapted to soft actuators, where integrating FBG becomes difficult and time-consuming. With the increasing development of disposable actuators in MIP, the requirement of cheap shape reconstruction methods is also essential. Due to the low cost of fabrication, they can be integrated into these devices where embedding EM and FBG sensors are not cost-effective. In this section, the different shape reconstruction processes using passive stretchable sensors have been briefly described, which is also summarized in Table 3.

**TABLE 3:** Summary of passive stretchable sensor-based shape reconstruction process.

Application	Author	Sensing principle	Method	Testing/medical Scenario	Evaluation
Soft actuator in MIP	SO et al. So et al. (2021)	9 skin-type stretchable resistive sensor on to 20 mm diameter and 200 mm length actuator	Strain value from sensor into Frenet-Serret formula	Free space	Tip tracking error as 4.45% of the length
Soft actuator	Cianchetti et al. Cianchetti et al. (2012)	10 pieces of conductive textile into the soft actautor	Strain values from sensor in the mechanical model	Free space	Shape reconstruction error varies between 3 and 6%
Soft actuator in MIP	Wurdemann et al. Wurdemann et al. (2015)	Three helically wrapped electro-conductive yarn as resistive sensors on to 30 mm long and 26 mm diameter STIFF-FLOP	Strain values in the kinematic model of actuator assuming constant curvature	Free space	Not reported
Variable stiffness manipulator used in MIP	Wei et al. Wei et al. (2020)	Low melting point alloy is used as resistance sensor in a 20 mm diameter and 133 mm long manipulator	Assuming constant curvature	Free space	The maximum angle error as -4.35°
Sensor-controlled antagonistic pneumatic actuators	Bilodeau et al. Bilodeau et al. (2018)	Conductive fabric as capacitive sensor on to soft actuator	Radius of curvature is calculated by calibrating sensor data with camera images	Free space	Close loop control of radius of curvature produced 2.8 $m^{-1}$

**FIGURE 5:**
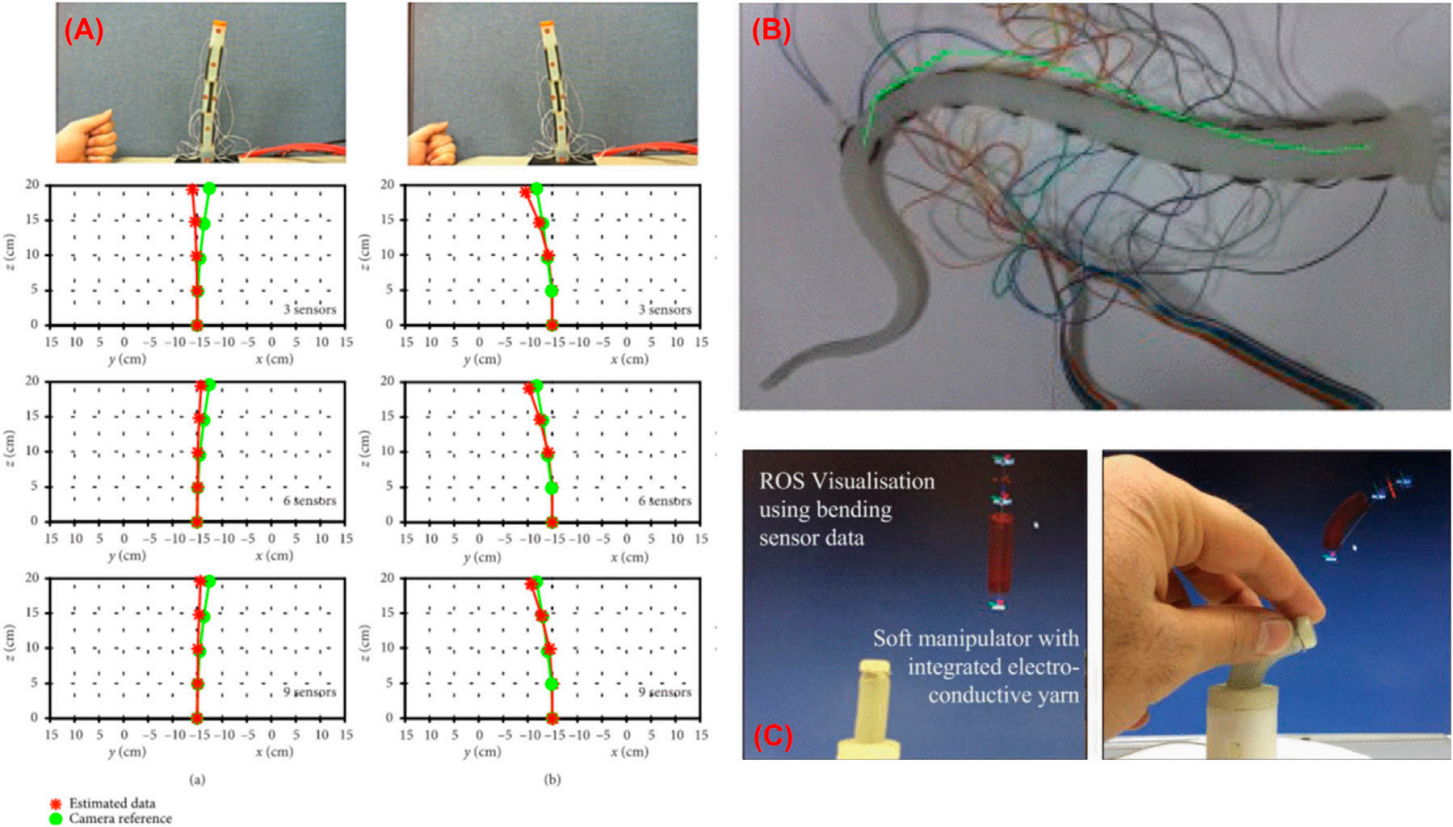
Shape reconstruction of soft manipulators **(A)** using skin stretchable resistive sensor (So et al., 2021), **(B)** using conductive fabric (Cianchetti et al., 2012), and **(C)** using electro conductive yarn (Wurdemann et al., 2015).

So et al. (So et al., 2021) proposed a method for reconstructing the shape of a soft manipulator used in MIP by implementing skin type stretchable sensors made from multiwalled carbon nanotube and silicone. The sensor used works on the principle of variable resistance, and the strain data from the sensor was utilized in the Frenet-Serret formula to reconstruct the shape (Figure 5A). When the reconstructed shape was compared with the camera data, it showed a tip position error of 4.45% of the total length. In another work, Cianchetti et al. (Cianchetti et al., 2012) presented a method that used stretch sensors to reconstruct the spatial configuration of an octopus-inspired robotic arm (Figure 5B). The stretch sensors work on the fundamental principle of resistance change and detect the local strain induced when deformations act on the arm. Data from the sensors were integrated into a dedicated model presented by Renda et al. (Renda and Laschi, 2012) and used to determine the arm’s curvature and shape. An error in the curvature evaluation was estimated, which varies between 3 and 6%. In another novel approach, Wurdemann et al. (Wurdemann et al., 2015) proposed a shape reconstruction process of a soft manipulator using shape sensors based on electro-conductive yarn (Figure 5C). Three sensors were embedded inside the soft silicon manipulator equipped with three pneumatic actuation chambers. The sensors worked on the principle of resistance change and were used to estimate the length of three chambers. The length information of these three chambers was utilized in dedicated equations to estimate the arc parameters of the manipulator. Abbas et al. (Abbas and Zhao, 2017) also presented another method that used a twisted and coiled sensor to estimate the shape of a soft robot. This sensor was made as twisted and coiled shape configuration using silver-coated nylon sewing threads. The principle of operation of this sensor is based on the change of resistance of the twisted and coiled sensor. Initially, a physical-based model was presented to estimate both the force and displacement of the sensor using resistance values. Afterward, the sensor was embedded into a soft silicone robot to estimate the curvature. A resistive sensor based on low melting point alloy (LMPA) was also used for reconstructing the shape of a variable stiffness manipulator intended to be used in MIP (Wei et al., 2020). In this case, the LMPA was encapsulated into the microchannel of a silicone tube and next, the tube was implemented with the manipulator. The change in resistance of the LMPA due to change in its cross-section area helped to measure the length of the silicone tube which was further used to find the bending information of the manipulator. Recently, the use of capacitive sensors for shape sensing has also been proposed in the literature. Bilodeau et al. (Bilodeau et al., 2018) presented a sensor-controlled antagonistic pneumatic actuator that employed a capacitance sensor fabricated using conductive fabric to reconstruct its curvature. When the actuator was controlled using the curvature information from the sensor, it produced an average error of 2.8 m^−1^.

Altogether, passive stretchable sensor-based shape reconstruction processes show many benefits such as low cost, high stretchability, and simple electronic circuits. Due to recent progress on skin type resistive sensors, they can be adopted into soft actuators used in MIP, avoiding complex sensor embedding processes. As they are intended to be made as stretchable, they are also used in the highly deformable soft actuators. Furthermore, due to low-cost manufacturing, this will reduce the cost of the disposable actuators with shape sensing technology. From the literature, it was observed that the shape reconstruction using resistive sensors is more suitable for soft robots and can be implemented on robots of different sizes (So et al., 2021), (Cianchetti et al., 2012). The current state of arts (Cianchetti et al., 2012) showed that the shape reconstruction error produced during these processes remains in between 3 and 6%. Though they are featured by many advantages, high shape reconstruction error and challenges faced during the wiring of multiple sensors limit their applications.

## Imaging-Based Shape Reconstruction Processes

Another field of research in shape reconstruction processes is imaging-based. These methods have the capability to measure the shape of robots without consuming extra space and requiring typically no major hardware modifications (Ren and Dupont, 2012). Image-based technologies like fluoroscopy (Papalazarou et al., 2012) (Hoffmann et al., 2012), endoscopy (Cabras et al., 2014), (Reilink et al., 2011) and ultrasound (Waine et al., 2015), (Carriere et al., 2016) are quite popular to estimate the shape of robotic manipulators. This section focuses on different intraoperative imaging techniques used for shape reconstruction of continuum robots, describing different methods and benefits, and addressing issues faced during the shape estimation process. The discussed medical image-based shape reconstruction applications are summarized in Table 4.

**TABLE 4:** Summary of medical imaging-based shape reconstruction process.

Application	Author	Imaging Modalities	Method	Testing/medical Scenario	Evaluation
Guide wire	Baert et al. Baert et al. (2003)	Biplane Fluoroscopy	Parametrization with epipolar geometry 2D tracking procedure based on energy minimization of spline	Intracranial anthropomorphic vascular	A mean distance to the reference standard of 0.42 mm
Catheter	Schenderlein et al. Schenderlein et al. (2010)	Biplane Fluoroscopy	B-snake algorithm with epipolar geometry	33 virtual images from patient data	Catheter pose error is 1.26 mm and mean tip deviation is 3.28 mm
Catheter	Hoffmann et al. Hoffmann et al. (2012)	Biplane Fluoroscopy	Graph search method with epipolar geometry	Clinical data	Mean error of robot shape is 0.4 ± 0.6 mm
Catheter	Hoffmann et al. Hoffmann et al. (2016)	Biplane Fluoroscopy	Graph search method with learning-based framework using epipolar geometry	Clinical and phantom data from cardiac intervention	Reconstruction error of 1.8 ± 1.1 mm on phantom and data and 2.2 ± 2.2 mm on clinical data
Snake like manipulator	Otaka et al. Otake et al. (2014)	Monoplane Fluoroscopy	Piecewise intensity based 2D/3D registration with prior knowledge of shape and kinematic property	hip osteolysis treatment	The joint angle error is 0.07°
Catheter	Papalazarou et al. Papalazarou et al. (2012)	Monoplane Fluoroscopy	non-rigid structure from motion (NRSfM) method	Moving the catheter in free space as well as inside a heart phantom model	Error not reported
Concentric tube robot	Vandini et al. Vandini et al. (2015)	Monoplane Fluoroscopy	Intra-operative tracking kinematic model of the robot using 2D/3D non rigid registration	A skull phantom	0.88 mm as shape reconstruction error and 2.22 mm as tip tracking error
Concentric tube robots	Lobaton et al. Lobaton et al. (2013)	Monoplane Fluoroscopy	Kinematics models with image data from optimal placed C-arm	A simulated lung phantom in bronchoscopy environment	The tip tracking error is 0.8 mm
Endoscopic instrument	Reilink et al. Reilink et al. (2011)	Endoscopy Imaging	Marker-less method which uses feature point information to update the kinematic model	A colon phantom in transluminal endoscopic surgery	RMS values for tip tracking error are 1.7, 1.2, and 3.6 mm for three directions
Endoscopic instrument	Reilink et al. Reilink et al. (2012)	Endoscopy Imaging	Marker-based: position information from maker used with kinematic model	Colon phantom in transluminal endoscopic surgery	RMS values for tip tracking errors are 2.3, 2.2, and 1.7 mm for three directions
Flexible instrument of an endoscope	Cabras et al. Cabras et al. (2014)	Endoscopy Imaging	Marker-based approach using supervised learning	*In vivo* images	RMS error of 1.64 ° 1.02, 1.52 ° 1.29, and 2.71 ° 1.52 mm respectively for x, y, and z coordinates
Needle	Greer et al. Greer et al. (2014)	2D-ultrasound	Using combined B-mode and power doppler images	Nitinol needle inserted along a curved path in *ex vivo* bovine liver tissue	Shape segmentation error 0.38 ± 0.27 mm
Needle	Carriere et al. Carriere et al. (2016)	2D-ultrasound	Combining a kinematic bicycle model with axial transrectal ultrasound (TRUS) image segmentation	*Ex-vivo* beef phantom tissue and *in vivo* clinical images	Tip prediction error of 0.497 ± 0.38 mm for *ex vivo* beef phantom tissue and 0.44 ± 0.15 mm for *in-vivo* clinical images
Needle	Waine et al. Waine et al. (2015)	2D-ultrasound	Intensity thresholding approach with Random Sample and Consensus (RANSAC) algorithm	Brachytherapy needle embedded within a tissue phantom	Shape estimation error 0.5 mm
Needle	Neshat et al. Neshat and Patel (2008)	3D-ultrasound	Bezier polynomial with generalized Radon/Hough transform	In an experiment testbed for robot assisted image-guided minimally invasive lung brachytherapy	Mean axis error for different needles: 0.48 mm for 18-gauge brachytherapy, 0.57 mm for 22-gauge brachytherapy

### Fluoroscopy-Based Shape Reconstruction Processes

Fluoroscopy is a medical imaging technique that uses ionizing radiation to produce two-dimensional images of the object. Typically, it consists of C-arms comprising of the X-ray source and photographic film faced opposite to each other. When an object is placed in between the source and film, the X-ray beam from the source is passed through the object and is captured by film, producing images of the interior parts of the object. This leads to a certain contrast on the image captured by film from which one may infer some anatomic structures or detect the shape of the instruments. Two kinds of fluoroscopy imaging processes exist that are biplane and monoplane, which will be described in this section along with their use in shape reconstruction procedures.

#### Biplane Fluoroscopy

**FIGURE 6:**
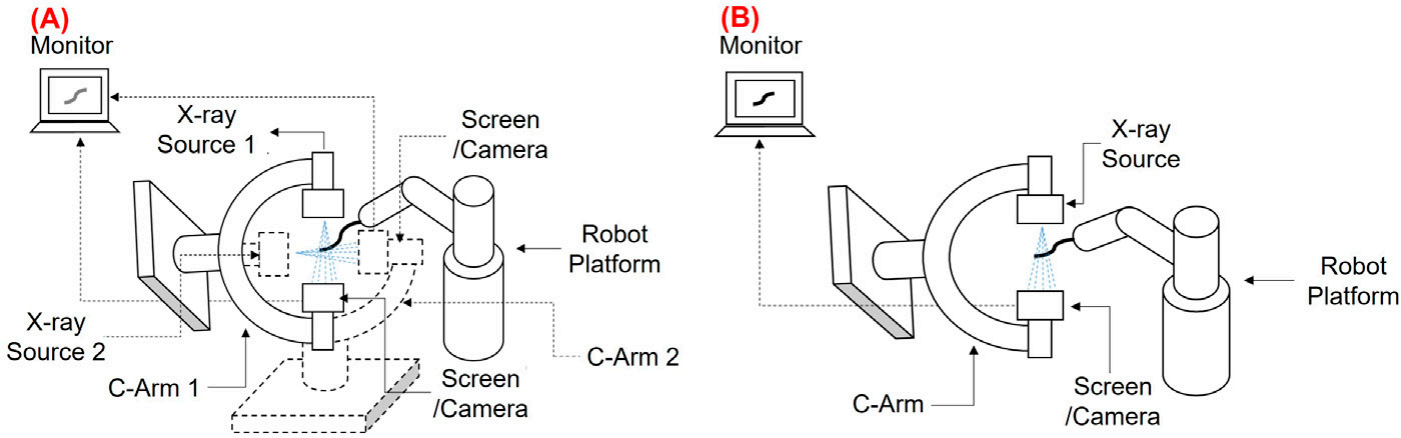
**(A)** Biplane fluoroscopy; **(B)** monoplane fluoroscopy.

In biplane fluoroscopy, two C-arms (Figure 6A) are placed independently around the object, and images are acquired simultaneously. This system synchronously provides two views of the object at different positions using both the C-arms. The first step towards fluoroscopy shape sensing is to acquire images from biplane fluoroscopy. The fluoroscopy images are then processed using various segmentation algorithms (Hoffmann et al., 2012), (Baert et al., 2003), (Schenderlein et al., 2010) to find out the device centerline. Finally, a 3D reconstruction method using epipolar geometry is implemented to find the 3D shape of the device.

**FIGURE 7:**
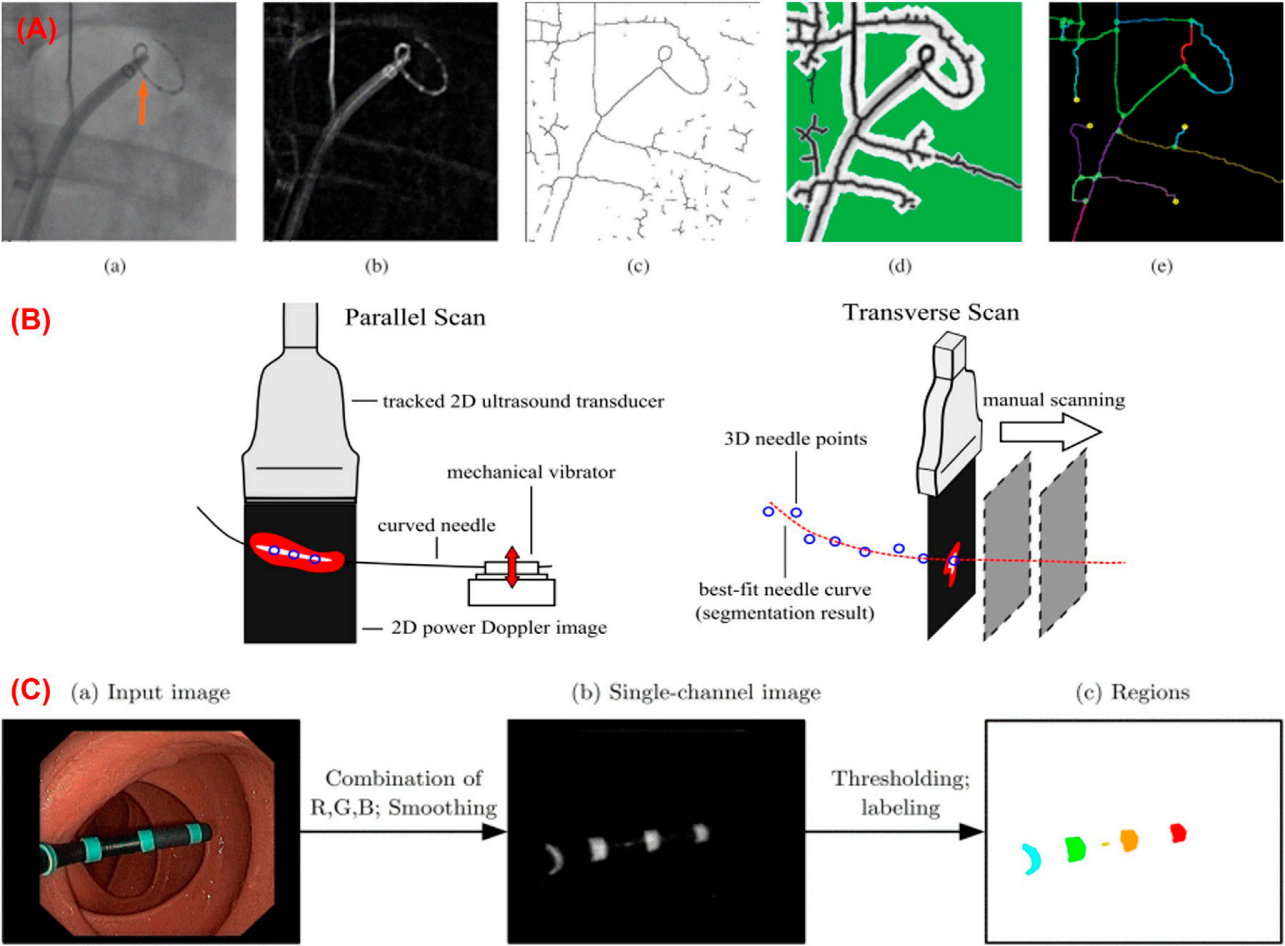
**(A)** Shape sensing using biplane fluoroscopy images (Hoffmann et al., 2016); **(B)** 3D shape reconstruction of a curved needle using ultrasound images (Greer et al., 2014); **(C)** Marker-based shape sensing using endoscopic camera images (Reilink et al., 2012).

Baert et al. used biplane fluoroscopy with epipolar geometry to reconstruct the 3D shape of a guidewire in the 3D vasculature (Baert et al., 2003). A 2D tracking procedure was implemented based on energy minimization of spline parametrization, where the guidewire was tracked and presented as a third-order B-spline line using acquired biplane images. Finally, the epipolar geometry was used to reconstruct the 3D shape of the guidewire from the biplane images using calibrated C-arm. Schenderlein et al. (Schenderlein et al., 2010) proposed a method that implements the B-snake (Menet, 1990) algorithm with biplane fluoroscopy images to reconstruct the 3D shape of a catheter. A line enhancing feature image was calculated and utilized to generate a 3D image from the 2D projections obtained from the biplane image system. To reconstruct the 3D pose of the catheter, missing image information caused by the asynchronous image acquisition was approximated by linear force interpolation using two consecutive images recorded by one of the C-arms. The evaluation of this algorithm was implemented on 33 virtual image datasets, and the mean catheter pose error was found as 1.26 mm. Hoffmann et al. proposed a method for reconstructing the 3D shape of a catheter using biplane fluoroscopy and epipolar geometry (Hoffmann et al., 2012). This approach performed three processes in which the catheter was first identified based on image pre-processing and then transformed into a graph to find an analytical representation model using the Dijkstra algorithm. In the last process, a final estimation of the 2D spline catheter was done using a search method. The catheter was then reconstructed into a 3D shape from the two 2D splines generated during the biplane fluoroscopy. The method was evaluated using 33 biplane images of a catheter. Since there are no 3D data available, the 3D reconstruction was forward projected into planes. The mean error was calculated as 0.4 ± 0.6 mm for the catheter. The error in 3D was calculated by acquiring 13 biplane images at different angulations (Hoffmann et al., 2013). The shape error was estimated as 1.2 ± 1.2 mm for the circumferential mapping catheter and 1.3 ± 1.0 mm for the ablation catheter. This method was further extended in (Hoffmann et al., 2016) by implementing a learning-based framework to adapt the arbitrary line-shaped catheter used in the Electrophysiology procedure (Figure 7A). This approach can be adapted to catheters that undergo larger deformations, such as coronary sinus catheters. The improved reconstruction error was estimated as 1.8 ± 1.1 mm and 2.2 ± 2.2 mm on phantom and clinical data.

#### Monoplane Fluoroscopy

Biplane fluoroscopy can be quite precise and consistent because it gives simultaneous views at various positions of the instruments to find out their shape. However, its applications are limited due to the high radiation exposure (Vandini et al., 2017), cost, and bulkiness of the instruments. To address these challenges, monoplane fluoroscopy was also proposed. The monoplane systems comprise only one C-arm (Figure 6B), thus reducing ionizing radiation dose in the shape estimation process. In this case, images are acquired from monoplane fluoroscopy and processed further with a segmentation algorithm to find the robot centerline. Finally, the shape of the robot is reconstructed using some kinematic models (Papalazarou et al., 2012).

Otaka et al. proposed a monoplane fluoroscopy-based shape reconstruction to generate the 3D shape of snake-like manipulators used in hip osteolysis (Otake et al., 2014). This approach used a piecewise intensity-based 2D/3D registration from an X-ray projection with prior knowledge of the shape and kinematic properties (e.g., each rigid structure connected by a pin joint parameterized by a low degree polynomial basis) for pose estimation. The feasibility of this method was tested using simulated projection images of the phantom of the cadaveric system and femoral hip implants simulating a scenario of treating osteolytic regions during hip revision surgery. The joint angle error was estimated as less than 0.07°. Papalazarou et al. presented a non-rigid structure from motion (NRSfM) method with a kinematic model to reconstruct a catheter (Papalazarou et al., 2012). This method used monoplane X-ray projection made with a small view separation for reconstructing the 3D shape of a deforming curvilinear catheter. The combination of NRSfM and the model provided a low dimensional parametrization of the catheter, which was then used with an X-ray imaging system to retrieve the 3D shape of the catheter. The suitability of this model was tested by moving the catheter in free space as well as inside a heart phantom model.

Vandini et al. used an algorithm that fuses information acquired from monoplane X-ray images with a kinematic model using 2D/3D non-rigid registration to reconstruct the real-time shape of the continuum robot (Vandini et al., 2015). The algorithm does not require repositioning of the C-arm, thus making it suitable for use in a constrained space. The method estimated 0.88 mm as shape reconstruction error. The same group also presented another method to reconstruct the 3D shape of concentric tube robots using a unified framework based on Markov random fields which combined the information from monoplane X-ray images with the kinematic model (Vandini et al., 2017). The reconstructed shape was compared with ground truth shape producing an error of 2.21 ± 1.1 mm. The methods described above are highly accurate and robust. However, these methods mainly rely on continuous exposure to ionizing radiation, which produces 2% of cancers due to the excess use of these imaging techniques (Brenner and Hall, 2007). To address this problem, Lobaton et al. proposed a new idea that used a small number of X-ray projection images to reconstruct the 3D shape of a robot (Lobaton et al., 2013). This approach first estimated the shape of the robot using a kinematic model and then refined the shape using data acquired from the monoplane X-ray projected images. To acquire the images, a method for optimal placement of C-arm was derived that extracted images at discrete time points. Although the method acquired discrete images at a certain point, it was capable of finding acceptable real-time shapes over the total procedure by integrating the previously extracted images with kinematics modeling. The method was implemented in a simulated bronchoscopy environment which produced a tip tracking error of 0.8 mm. Trivisonne et al. (Trivisonne et al., 2020) proposed an approach that combined a physical-based model with a non-linear Bayesian filter for reconstructing the shape of a catheter in a 3D workspace. Initially, the model predicted the shape of the catheter, and then it was corrected by Unscented Kalman Filter using 2D single-view fluoroscopy images. The reconstructed shape was compared with the known ground truth producing a 3D Hausdorff Distance of 0.81 ± 0.53 mm for the synthetic data set and 1.77 ± 0.77 mm for the real data set.

As MIP primarily focuses on smart flexible robots which can autonomously propel through complex deformable tubular structures, shape reconstruction is essential to accurately control the robot without damaging the environment. In this case, electromagnetic shape reconstruction may fail in the presence of other electrical components, and FBG-based shape reconstruction methods may not be implemented due to the highly complex deformable structure of the robot. As fluoroscopy techniques are free from electromagnetic interferences and the calculation of strains, this technique remains a promising method for reconstructing the shape of these highly deformable robots accurately. Despite these advantages of fluoroscopy imaging technology, limitations like the use of bulky and costly instruments and larger ionizing radiation dosage remain a concern. Methods, whereby different reconstruction modalities should be fused which could provide the best reconstruction process while reducing the total radiation exposure. Further research should be done to use this technique safely in MIP targets.

### Ultrasound-Based Shape Reconstruction Processes

Fluoroscopy-based imaging processes are able to estimate the shape of flexible instruments and robots in MIP pretty accurately without requiring any extra sensors in the robot. However, the use of high radiation dosage and dependence of on nephrotoxic contrast agents limit them in shape reconstruction processes due to safety issues. Another low-cost and safe imaging modality that can be used to see through tissue and some obstacles is ultrasound. This sensing modality is surveyed in this section.

Ultrasound imaging technology is another alternative to estimate the shape and track the instruments without exposure to large radiation dosages. Although the images produced in ultrasound lack good visibility (Cheung and Rohling, 2004), this process could be used to reconstruct the shape consistently. In addition to this, ultrasound imaging is safe, fast, portable, and economical. This method has been implemented to detect the position of medical instruments (Novotny et al., 2006), (Novotny et al., 2007) to determine the position and orientation of long, thin surgical tools (Uherčík et al., 2010), to estimate needle deflection or track the tips of steerable needles (Wei et al., 2004), (Zhao et al., 2012) and to detect continuum curved robot (Ren and Dupont, 2012) in different medical scenarios.

Greer et al. (Greer et al., 2014) presented a real-time segmentation method to reconstruct the 3D shape of curved needles using combined B-mode and power Doppler images from a tracked 2D ultrasound transducer (Figure 7B). The approach consisted of image analysis and curve fitting steps. In the first step, 2D points along the needle were generated using the image analysis on the pairs of power doppler and B-node images. Subsequently, the 2D points were transformed to 3D world coordinates, which were further used for the 3D shape reconstruction process. When this method was compared with manual segmentation of the needle (identifying the needle in each image manually and fitting a 3D polynomial to the identified points), it produced an error of 0.38 ± 0.27 mm in the shape reconstruction process.

Carriere et al. proposed a real-time method to estimate the shape of a flexible needle by combining a kinematic bicycle model with axial Transrectal Ultrasound (TRUS) image segmentation (Carriere et al., 2016). In this method, the location of the cross-section of the needle was segmented out of each of the image slices captured normally to the insertion direction of the needle using ultrasound imaging technology. A particle filter incorporated the tracked cross-section and the distance between successive image slices to update the parameters of the kinematic model for each image slice. The parameters of the model were updated up to a pre-defined insertion depth and then the model predicted the shape of the needle for complete insertion. The method was tested in *ex-vivo* beef phantom tissue and *in-vivo* clinical images and produced an average tip prediction error of 0.497 mm ± 0:38 mm (pre-defined depth 60 mm) and 0.44 ± 0.15 mm (pre-defined depth 35 mm). respectively. Waine et al. also employed 2D transverse ultrasound images to visualize the 3D shape of the needle used in the brachytherapy process (Waine et al., 2015). They applied an intensity thresholding approach to find out the potential locations of the needle within each 2D transverse ultrasound image. A Random Sample and Consensus (RANSAC) algorithm was then implemented to filter out the outliers, and then remaining points were fitted in a polynomial model to reconstruct the 3D shape of the needle. The method was validated using 21 sets of ultrasound images of the brachytherapy needle embedded within a tissue phantom, and the estimated shape of the needle differed 0.5 mm with respect to shape measured using a camera. Three images from an insertion depth 50 mm or greater predicted the entire shape.

Shape sensing using 3D ultrasound imaging has also received much attention in MIP. Neshat et al. proposed a novel method to detect the needle in real-time using 3D ultrasound images (Neshat and Patel, 2008). They presented an algorithm based on parameterization of the shape of the instrument (needle) using Bezier polynomials and the generalized Radon/Hough transform for real-time detection of the needle. This algorithm was implemented on a graphics processing unit (GPU) using Compute Unified Device Architecture (CUDA) programming. The effectiveness of the method was tested in an experimental testbed for robot-assisted image-guided minimally invasive lung brachytherapy in which the mean axis error remained near to 1 mm when compared with EM trackers. Ren and Dupont (Ren and Dupont, 2012), (Ren and Dupont, 2011) introduced a new method to detect the curved robot using the 3D ultrasound imaging technique. This method fused geodesic active contours and a speed function based on the enhancement of tubularity of the robot for the detection. The proposed method was tested in *ex vivo* intracardiac experiments. Although shape sensing using 3D ultrasound imaging is fast and accurate, the cost of 3D ultrasound systems as compared to 2D ultrasound devices is higher which limits the successful implementation of this 3D ultrasound method (Waine et al., 2015).

Despite many advantages of fluoroscopy shape sensing techniques, such as being fast, accurate, free from EM interference, these methods generally use high radiation dosage and limit the application due to safety issues. Ultrasound imaging can be an alternative to fluoroscopy to estimate the shape of a robot without exposure to ionizing radiation. This method is safe and free from line-of-sight and is generally used for sensing the shape of the needle used in MIP intervention. In addition to this, ultrasound imaging techniques can also be used for estimating the shape of continuum robots accurately. As MIP deals mainly with continuum deformable robots, this method can be implemented for the shape reconstruction of robots. While this method shows a lot of advantages, low resolution and signal-to-noise ratio are challenges of ultrasound-based shape reconstruction. Further research in this field is crucial for adopting this technology in applications targeted by MIP.

### Endoscopic Camera-Based Shape Reconstruction Processes

Unlike fluoroscopy imaging and ultrasound, endoscopic camera-based shape reconstruction uses the images of the instrument from an endoscopic camera to reconstruct its shape. The shape reconstruction procedure using endoscopes typically follows two approaches. The first approach is called marker-less, in which different feature points are extracted from the images of the instrument taken by the endoscopic camera. These feature points are then used further to find the shape of the instrument. The second approach is marker-based, in which the shape of the instrument is reconstructed by tracking the markers present on it. These approaches are further divided into different categories based on the type of model used, i.e. kinematic model-based and model-free approaches, and the type of instruments used, such as traditional or robot-assisted instruments. In the following section, an overview is given of reconstruction techniques based on these modalities.

Recently Sestini et al. (Sestini et al., 2021) proposed a marker-less self-supervised image technique based on the kinematic model to estimate the pose of the surgical instruments of a robotic endoscope. In this case, a regressor model was trained to determine joint values of the instrument using the camera images, and then these values were fused with the forward kinematic model to reconstruct its 3D shape. The validation of the presented method was done in three different data set consisting of real-time acquisition (phantom and *in-vivo*) and a semi-synthetic. For the semi-synthetic data set, when the reconstructed shape was compared with the known ground truth, the mean absolute translation and bending error were estimated as 1.75 and 0.47 mm for the left instrument and 1.17 and 0.30 mm for the right instrument, respectively. For phantom and *in-vivo* datasets, the reprojection error was calculated with respect to manually annotated ground truth via insertion over union which showed 0.64 (phantom), and 0.55 (*in-vivo*) for the left instrument and 0.725 (phantom) and 0.554 (*in-vivo*) for the right instrument. Reilink et al. also proposed a marker-less approach to reconstruct the 3D pose of an instrument of an advanced flexible endoscope (Reilink et al., 2011) using a kinematic model. The images from the endoscopic camera were processed to find the feature points on the instruments. These features were compared with the calculated position of these points acquired from the kinematic model. Depending on the deviation between the model and observations, the constructed state of the model was updated repeatedly so as to match the model with observations. The same group extended the study and presented marker-based approaches by placing four markers on the instrument (Figure 7C) (Reilink et al., 2012). In this approach, instead of feature points, the position information from the four markers was used to update the kinematic model of the instrument. The approaches were tested inside a colon model. The marker-based approach produced RMS errors of tip tracking as small as 2.3, 2.2, and 1.7 mm, while the marker-less approach produced an RMS error of 1.7, 1.2, and 3.6 mm along the X (horizontal), Y (vertical) and Z (away from the camera) directions, respectively. Both methods require precise geometry and kinematic models of the robot. However, obtaining such precise geometry may not be practical, and sometimes they do not produce satisfactory accuracy due to uncertainty in the model parameters (Cabras et al., 2014). Cabras et al. presented an improved marker-based approach using supervised learning with prior knowledge of the kinematic model of the instrument of an advanced endoscope for shape reconstruction (Cabras et al., 2014). This method reduced the uncertainties produced by previous approaches. With the support of colored markers fixed to the instruments, this method used an image segmentation stage followed by a stage for position estimation. In the beginning, the markers were segmented using an Adaptive Boosting algorithm trained by manually determining foreground and background samples from *in-vivo* images taken during operations. Thereafter, the centroid of the markers was calculated and used as input to the data pose estimation stage.

With disposable chip-on-tip cameras becoming available, endoscopic camera-based shape reconstruction processes could be implemented in MIP to avoid bulky and costly instruments. This technique uses robot cameras to estimate the shape, thus eliminating the use of any external instrument and high radiation dosage. As this technique is successfully used for highly deformable continuum robots, it can be implemented in MIP targets to estimate the shape of the robot accurately. This can also reduce the space of the workplace by eliminating larger size instruments which could be a great advantage in MIP where the main aim is to optimize the reachable workspace. However, a direct line-of-sight is required in this process to estimate the shape of the robot. Obstructions or occlusions in this process will create problems in shape estimation. Further advancement in this research is essential to implement this concept, possibly requiring fusion with other sensor modalities.

In summary, medical imaging technologies such as fluoroscopy and ultrasound remain a standard procedure for visualizing the shape of continuum manipulators in MIP till date despite the advancement of other shape reconstruction processes. Most of the clinical personnel rely on these methods as they involve direct visualization without the requirement of calculation strain and other complex processes. As there is no involvement of internal sensor, avoiding requirements of stretchable sensor and hardware modifications. These methods are also less dependent on temperature effects at room conditions, making them suitable to be implemented in most robotics platforms. As there is no involvement of sensors, they can be used in many continuum manipulators regardless of their size. While owing many advantages, these techniques have some challenges to deal with, such as large radiation dose and dependence on nephrotoxic contrast agents in case of fluoroscopy, low resolution, and signal-to-noise ratio of ultrasound images and obstructions in front of endoscopic cameras. Another drawback is the use of bulky instrumentation which limits their application in space constraint clinical environments.

## Discussion

Continuum and flexible robotic systems have been widely considered to facilitate complex medical interventions in minimally invasive surgeries for safer, comprehensive, and more reliable procedures. As the robots used in these processes produce a great range of flexibility and maneuverability, shape sensing, and tip tracking remain the gold standard for controlling them more accurately inside the anatomy. This study reviewed the development, technical advancement, and state of the art of different shape reconstruction methods used for estimating the shape of the robot in MIP. Shape reconstruction methods using sensors such as fiber optics, position-based, and passive stretchable sensor-based and imaging techniques such as fluoroscopy, endoscopic camera, and ultrasound were investigated.

Fiber optic-based shape reconstruction is frequently employed due to its advantages, such as small size, flexibility, and immunity to EM interference. They can be classified into four groups according to the detecting the modulation of the light which are intensity, phase, scattering, and wavelength. The first three methods are generally used to detect single bending modes of the continuum robots. However, in continuum robots, since multi bending modes combined with torsion are common, this review focuses on reconstructing curvature by measuring wavelength shift. FBG-based sensors are a wide class of applications of this method. They are highly sensitive, have a short response time, suitable for high-frequency applications, and have the potential to reconstruct the shape of the robot with sub-millimetric error. However, the cross-sensitivity to strain and temperature and low stretchability of the FBG sensor remain drawbacks which are needed to be considered during applications. To overcome these limitations, various solutions have been proposed. For example, the cross-sensitivity to the temperature has been eliminated by employing extra FBG sensors which are not affected by strain. However, it makes the design complex and increases the cost to the system. On the other hand, to detect large curvature, which is higher than the stretchability of an FBG, the fiber with FBG sensors was coupled with superelastic wires (e.g., Nitinol), aiming to reduce the induced strain on the FBG due to the bending. Although it allows for detecting large curvatures, the reconstruction error increases.

Position-based sensors offer significant advantages such as miniaturization, high sensitivity, freedom from line-of-sight, and high accuracy. These benefits of the sensors help to incorporate inside the small and highly flexible catheters, endoscopes, and other medical instruments to reconstruct their shape. The shape of the instruments is estimated by using tracking data with kinematic models or position data with the Bezier curve. In some cases, only using a few numbers of position sensors, such as using a single sensor at the tip or using two sensors i.e., one at the tip and another at the base, the shape of the instruments is estimated. However, such approaches do not produce qualitative data due to the lack of ground truth and the use of highly discrete data. These issues can be solved by placing more sensors along the instrument and incorporating sensor data with other imaging technologies to generate quality ground truth and shape reconstruction information. Keeping aside the benefits, they also produce a major drawback, such as tracking results being compromised due to the presence of ferromagnetic materials. The other shortcomings, such as EM interference due to the presence of other magnetic fields in the working environment and the adoption of interpolation techniques to reconstruct the shape due to the discrete pose information produced from these methods limit their robustness. Some methods using EM tracking information with other sensing modalities such as combining EM data with fluoroscopy images and other predictive models have been used to counteract such disturbances. Different approaches such as using redundant EM sensors are developed to improve the accuracy of these methods. Although these approaches show propitious results, further research can help to make them more reliable and sturdier.

Shape reconstruction using medical imaging techniques such as fluoroscopy, endoscopic camera, and ultrasound estimate the shape of the flexible manipulator accurately and precisely without consuming extra space and no major hardware modifications. Such techniques produce more accurate and real-time shape information as they do not deal with the calculation of strains and electromagnetic interference. In some approaches, biplane fluoroscopy images with epipolar geometry are employed to estimate the shape. Since biplane fluoroscopy produces simultaneous views of the position of the instrument, these approaches show precise, accurate, and consistent shape reconstruction information. Despite these benefits, the biplane fluoroscopy imaging system produces significant drawbacks such as high radiation dosages, expensive and bulky instruments, and difficult to use in a constrained operating workspace. To deal with these shortcomings, some other methods using monoplane fluoroscopy images with kinematic models have been evolved to reconstruct the shape of medical instruments. Though these approaches resolve some of the shortcomings of biplane fluoroscopy, continuous exposure to ionizing radiation creates problems during successful implementations. In a new approach, the continuous exposure to ionizing radical is reduced by using a few numbers of X-ray projection images along with a kinematic model to reconstruct the shape. Despite the reduction of exposure to the ionization, they may produce significant error due to the use of discrete data from optimal placement of C-arm.

Other alternatives of shape reconstruction approach using ultrasound images and endoscopic images have been evolved to deal with the issues produced by fluoroscopy imaging systems. Even though these techniques produce significant advantages with respect to fluoroscopy imaging systems such as elimination of bulky and expensive instruments, contrast agent and exposure to large radiation dosage, they need to deal with low signal to noise ratio in the case of ultrasound images and obstruction or occlusion in front of the endoscopic camera. Therefore, further technical advancement and improvement in the medical imaging techniques are highly crucial to provide a safe, accurate, low-cost, and trouble-free shape reconstruction method.

One of the major drawbacks of FBG, EM, and medical imaging shape reconstruction processes such as fluoroscopy and ultrasound is high cost. With the increase in demand for disposable instruments in MIP, the use of these technologies may not be cost-effective and efficient. In this case, the passive-stretchable sensor-based shape reconstruction processes can play a major role in estimating the shape of the robots. Other problems like the stretchability issue of FBG can be resolved with this solution. Despite these advantages, the high shape estimation error and wiring issue remains a concern and drawing attention to work more on this field to develop a highly accurate shape reconstruction process.

The qualitative comparison between each shape reconstruction method is presented in Figure 2 considering all the aspects. It showed that passive-based and endoscopic camera shape reconstruction processes are good for highly deformable manipulators. They are also low-cost shape reconstruction methods as they involve low-cost fabrication methods in the case of passive stretchable sensors and the use of the previously present cameras in the case of endoscopic camera imaging. Sensor-based technologies using FBGs and imaging technologies such as fluoroscopy and ultrasound can be implemented to small manipulators as FBGs are small and imaging methods process images without modification of manipulator. The position-based sensor can be integrated into a highly flexible manipulator with moderate accuracy and miniaturization. The selection of shape reconstruction process can be made based on involved devices and the requirement criteria, however, the associated problems which are described in this section should be considered.

In summary, more than a hundred research articles have been published recently which discussed position sensor-based and FBG sensor-based shape reconstruction techniques. EM position-based shape reconstruction is helpful if the target instruments and working environments deal with non-ferromagnetic materials. The size of FBG sensors makes them of interest for implementation in small medical instruments such as active biopsy, as well as trocar needles, and steerable catheters. Though these processes have been rapidly developing with the advancement of medical devices, open questions have to be solved before having them employed in clinical applications. The first question is about their reliability, as these processes only result in model-based simulated shape unlike the direct visualization produced during medical imaging techniques such as fluoroscopy and ultrasound. A second question is how much error in the reconstruction processes is acceptable for handling the instruments safely inside the anatomy.

As some clinical applications involve the use of disposable instruments, the use of these processes (EM and FBG) is not cost-effective when expensive sensory systems are being considered. In the case of reusable instruments, these systems can still be cost-effective. However, the sterilization of the instruments may lead to breakage or faulty data due to sensor property modifications. In this case, there are also emerging questions such as how many cycles they can sustain before breaking or providing incorrect measurements and how to solve the complexity in sensor embedding processes.

Passive stretchable sensor-based shape reconstruction processes are still at in an early stage of development. They need many technical advancements to enter clinical environments such as issues of biocompatibility, wire integration, material fatigue, and hysteresis issues are yet to be solved. Other limitations such as effective ways to perform integration into clinical devices and selection of optimal sensor number and location remain unresolved. These issues lead to a query, i.e., how will these techniques evolve in the future that will not only enhance the robustness but also accelerate their use in clinical applications.

Image-based techniques have been widely used in clinical applications thanks to their direct visualization and the joint use for traditional diagnostic imaging. Efforts have been made using different predictive and learning algorithms to estimate the shape. However, a question emerges naturally: how efficient are these techniques when dealing with obstruction, noise, and occlusion? In the future, these approaches may involve other models or calibration processes to deal with these issues. In any case, they should be improved in such a manner that will be straightforward to use without the direct intervention of users.

## Conclusion and Future Directions

In this review, papers proposing unique shape reconstruction processes employed in devices for medical interventions were discussed. The motivation of this review was to describe and qualify the existing shape reconstruction processes to the readers. Initially, a systematic categorization was performed where the shape reconstruction field was divided into two main parts i.e., sensor-based and image-based. Next, the sensor-based techniques were divided into three subcategories based on the types of sensors used, and the image-based techniques were also classified into three subgroups according to the use of image processing methods. Thereafter, the working principle, and the algorithm involved, as well as the method details and the advancement in each classification were reviewed. To the end of each category, its benefits and limitations were also discussed.

In this analysis, it was observed that each technique has its advantages and limitations. Hence, it can find a useful application in a certain condition. Thus, the idea of a hybrid shape reconstruction process is an emerging research field nowadays which fuses the features from more than one sensing strategy. Hybrid solutions have been developed to combine the advantages from each solution to generate more robust measurements and covering broader environments. For example, one system which combines fluoroscopic imaging technique with position sensing (EM and permanent trackers) can be used in two different environments i.e. when dealing with electromagnetic interference where only implementing EM sensing leads to loss of accuracy and another when dealing with a high risk-prone area where the use of the fluoroscopic solutions is unsafe to use. In the case of FBG sensor-based reconstruction processes, it is hard to distinguish twist from strain, which leads to increase in shape reconstruction error. Different techniques using fluoroscopic system combined either with FBG sensing or with both position and FBG sensing can be proposed to include the twist information. There are few papers (Ourak et al., 2021), (Ha et al., 2021) that proposed these kinds of strategies. But further development is necessary to bring these systems into the medical scenarios. Another interesting reconstruction solution could be fusing the shape information from position sensors with ultrasound imaging. Initially, the shape of the instrument could be reconstructed using position information from the EM sensor in the Bezier curve and refining the shape by using ultrasound images. As ultrasound imaging technique generates actual shape information of instrument, this may help to reduce the error produced during interpolation method used in position-based shape reconstruction process. Other novel sensing solutions could be developed by combining position-based shape sensing approaches with FBG sensor data. This may be implemented in high risk-prone areas where medical imaging system such as fluoroscopy is highly unsafe and space constraint makes difficult the use of bulky instruments. Some of these hybrid solutions can also answer few open questions discussed in this paper. Hybrid solutions that involve ultrasound and fluoroscopy may solve the issue of reliability produced during the shape reconstruction process using FBG and EM sensors Though these techniques will increase the robustness of the shape reconstruction processes, they might face some challenges such as dealing with different refresh rates, problems of synchronization, system complexity, and high cost and needs to be considered for developing a new hybrid system during research.

Force sensing in the case of the device involved in medical interventions is also a growing interest of researchers apart from shape reconstruction processes for reducing tissue damage and increasing safety. However, integrating force sensors onto these devices is very complicated and costly. In this case, FBG based sensors can be used to provide the force and shape information simultaneously without requiring extra sensors and might achieve greater control by providing multiple feedback to the system. Different groups proposed methods which use various models (Roesthuis and Misra, 2016; Qiao et al., 2021) with FBG sensor data for finding shape and force. However, this area has not been fully explored and needs further advancement.

Future directions can also include the development of an isolated/shielded environment to deal with EM interference coming from the surroundings which may increase the accuracy of position-based shape reconstruction processes. The current era is an era of machine learning, deep learning, artificial intelligence. Thus, these methods can be employed in the case position-based shape reconstruction process which may reduce the error of interpolation by learning from the previously acquired shape reconstruction data.

The passive stretchable sensor-based shape reconstruction processes are gaining popularity due to their low-cost fabrication process and high stretchability. However, they produce high shape reconstruction error due to the hysteresis behavior of the involved material. Thus, this remains unsolved and can be an active area to direct research efforts in the future. The strain sensors based on variable electrical inductance principle recently got attention due to their high accuracy and low hysteresis (Azami et al., 2019), (Xing et al., 2020). They could represent a step forward in this direction.
